# Multiple cross-frequency coupling analysis of resting-state EEG in patients with mild cognitive impairment and Alzheimer’s disease

**DOI:** 10.3389/fnagi.2023.1142085

**Published:** 2023-08-04

**Authors:** Xi Chen, Yingjie Li, Renren Li, Xiao Yuan, Meng Liu, Wei Zhang, Yunxia Li

**Affiliations:** ^1^School of Communication and Information Engineering, Shanghai Institute for Advanced Communication and Data Science, Shanghai University, Shanghai, China; ^2^College of International Education, Shanghai University, Shanghai, China; ^3^School of Life Science, Institute of Biomedical Engineering, Shanghai University, Shanghai, China; ^4^Department of Neurology, Tongji Hospital, School of Medicine, Tongji University, Shanghai, China; ^5^Department of Neurology, Shanghai Changhai Hospital, the Second Military Medical University, Shanghai, China; ^6^Department of Neurology, Shanghai Pudong Hospital, Fudan University Pudong Medical Center, Shanghai, China

**Keywords:** mild cognitive impairment, Alzheimer’s disease, resting state, EEG, cross-frequency coupling, phase-amplitude coupling

## Abstract

**Introduction:**

Electroencephalographic (EEG) abnormalities are seen in patients with Alzheimer’s disease (AD) and mild cognitive impairment (MCI) with characteristic features of cognitive impairment. The most common findings of EEG features in AD and MCI patients are increased relative power of slow oscillations (delta and theta rhythms) and decreased relative power of fast oscillations (alpha, beta and gamma rhythms). However, impairments in cognitive processes in AD and MCI are not sufficiently reflected by brain oscillatory activity in a particular frequency band. MCI patients are at high risk of progressing to AD. Cross-frequency coupling (CFC), which refers to coupling between different frequency bands, is a crucial tool for comprehending changes in brain oscillations and cognitive performance. CFC features exhibit some specificity in patients with AD and MCI, but a comparison between CFC features in individuals with these disorders is still lacking. The aim of this study was to explore changes in CFC properties in MCI and AD and to explore the relationship between CFC properties and multiple types of cognitive functional performance.

**Methods:**

We recorded resting-state EEG (rsEEG) signals in 46 MCI patients, 43 AD patients, and 43 cognitively healthy controls (HCs) and analyzed the changes in CFC as well as the relationship between CFC and scores on clinical tests of cognitive function.

**Results and discussion:**

Multiple couplings between low-frequency oscillations and high-frequency oscillations were found to be significantly enhanced in AD patients compared to those of HCs and MCI, while delta-gamma as well as theta-gamma couplings in the right temporal and parietal lobes were significantly enhanced in MCI patients compared to HCs. Moreover, theta-gamma coupling in the right temporal lobe tended to be stronger in MCI patients than in HCs, and it was stronger in AD than in MCI. Multiple CFC properties were found to correlate significantly with various cognitive domains, especially the memory function domain. Overall, these findings suggest that AD and MCI patients must use more neural resources to maintain a resting brain state and that alterations in theta-gamma coupling in the temporal lobe become progressively obvious during disease progression and are likely to be a valuable indicator of MCI and AD pathology.

## 1. Introduction

Alzheimer’s disease (AD) is a progressive neurological disorder with an insidious onset (Scheltens et al., 2021) and is the most common type of dementia (Alzheimer’s Association, 2022). Mild cognitive impairment (MCI) is a transitional stage between healthy aging and dementia (Petersen, 2016), individuals with MCI represent a potential onset population for AD and have a much higher probability of developing AD than normal people (Manly et al., 2008; Alzheimer’s Association, 2022). In contrast to cognitively normal older adults, the hallmark clinical symptoms of AD patients are difficulties with memory, language, problem-solving and other thinking skills (Alzheimer’s Association, 2022), while MCI patients may suffer cognitive impairments highlighted by memory impairment and mood regulation disorders (Petersen, 2016). Numerous factors that reflect specific changes in the brain are responsible for these symptoms, including the accumulation of the protein beta-amyloid (plaques) outside neurons, twisted strands of the protein tau (tangles) inside neurons in the brain, inflammation, atrophy and massive neuronal and synaptic loss in the cerebral cortex and hippocampus (Jafari et al., 2020; Alzheimer’s Association, 2022). The development of neurophysiology has made it possible to measure these brain changes and assist clinicians in making clinical diagnoses. For example, positron emission tomography (PET) can detect accumulation of beta-amyloid and tau protein (Alzheimer’s Association, 2022), whereas structural Magnetic Imaging (sMRI) can show medial temporal lobe atrophy (Jack et al., 2018), and so on. Despite this, the practical utility of these neuroimaging techniques continues to be restricted by high infrastructure costs (Farina et al., 2020). Among existing methods, electroencephalography (EEG) can directly reflect brain electrical activity in real time at the synaptic level (Jafari et al., 2020; Rossini et al., 2020), while synaptic damage and loss has are fundamental to the pathophysiology of AD and lead to reduced cognitive function (Colom-Cadena et al., 2020). Moreover, taking into account its convenience, widespread availability, non-invasiveness, EEG analysis may be an excellent candidate for tracking the prodromal phases of cognitive decline in routine clinical settings (Rossini et al., 2020).

Scalp resting-state EEG (rsEEG) is a state in which the brain is not engaged in any particular cognitive task and is instead quiet, relaxed and awake. The rhythms of rsEEG reflect the summation of oscillatory membrane post-synaptic potentials generated from cortical pyramidal neurons, which play the role of EEG sources (Rossini et al., 2020) and can establish the baseline condition of the brain and provide a control for task-state investigation (Kan et al., 2017). Correlational studies suggest that abnormalities of cortical rsEEG oscillations are linked to both volumetric cortical neurodegeneration and cognitive test scores in patients with MCI and AD, and different rsEEG rhythms have been extensively studied in pathological brain aging (Hamm et al., 2015; Hata et al., 2016 Jafari et al., 2020). According to previous studies, AD and MCI patients are characterized by an increase in the relative power of slow oscillations (delta and theta rhythms) and a decrease in the relative power of fast oscillations (alpha, beta and gamma rhythms) compared to age-matched healthy control subjects (Hamm et al., 2015). However, it is noteworthy that brain rhythmic activities in different frequency bands are not isolated (Buzsáki and Watson, 2012). Cross-frequency coupling (CFC), which describes the statistical relationship between the amplitudes, phases, and frequencies of two distinct frequency bands, lies at the heart of our understanding of the communication and integration that occur between nearby or distant neuronal groups (Canolty and Knight, 2010) and plays a pivotal role in exploring the neural mechanisms that underlie psychiatric disorders.

A growing body of research has revealed that CFC plays a crucial functional role in working memory and cognition as well as in AD and MCI (Yakubov et al., 2022). For example, the significantly increased CFC in amnestic MCI (aMCI) patients was reported to correlate with task performance during a standard auditory oddball paradigm, and theta-gamma coupling was found to identify aMCI patients with 95% accuracy (Dimitriadis et al., 2015). In a study that assessed the relationship between theta-gamma coupling and working memory deficits in AD and MCI patients, AD patients exhibited the lowest level of theta-gamma coupling when the subjects performed 1- and 2-back working memory tasks, followed by MCI patients and healthy controls (Goodman et al., 2018). By measuring theta-gamma coupling in AD and aMCI patients during their performance of an olfactory task, Fatemi et al. (2022) discovered significant differences in coupling among AD, aMCI and healthy controls that help explain the underlying processes involved in cognitive impairment as well as the observed differences in the performance of cognitive tasks by aMCI and AD patients. It appears that in the analysis of CFC detected by task-state electroencephalography (EEG) in AD and MCI patients, the coupling between theta and gamma rhythms has become the focus of interest.

It is worth noting that the aforementioned studies are based on task-state EEG. However, experimental data collection during performance of a task is fraught with inconveniences, especially in the study of AD patients, and there is considerable evidence for the effectiveness of rsEEG as a non-invasive predictive biomarker of neurodegeneration and of the transition from MCI to AD (Jafari et al., 2020; Babiloni et al., 2021); thus, many researchers prefer to use rsEEG when studying the brains of AD patients. To date, only a handful of studies have investigated CFC alterations during rsEEG in individuals with AD or MCI. Wang et al. (2017) reported enhanced coupling between beta/gamma and low-frequency bands in AD patients and speculated that this enhanced coupling may be caused by interneuronal dysfunction of GABAergic neurons. Conversely, Poza et al. (2017) found a significant reduction in alpha-gamma coupling in the left inferior temporoparietal lobe in AD patients. A recent study by Vanneste et al. (2021) found that theta-gamma coupling between the posterior cingulate cortex and the left and right parahippocampal regions may be responsible for impaired memory function in patients with aMCI. We also note that most previous studies have addressed only one type of disorder, either AD or MCI. It has been shown that approximately 15% of MCI patients progress within 2 years to more severe states of dementia and even to AD and that 2/3 of AD patients have been previously diagnosed with MCI (Alzheimer’s Association, 2022), suggesting that the majority of MCI patients may have underlying AD pathology. Therefore, it is important to investigate how coupling characteristics change along the continuum from subjective cognitive decline to MCI and ultimately to AD. To do this, it is necessary to compare the characteristics of patients with MCI to those of patients with AD. To date, only the study by Musaeus et al. (2020) has examined coupling differences in rsEEG in AD, MCI and healthy controls (HC) concurrently. That study reported a non-significant reduction in theta-gamma coupling in AD patients and a significant positive correlation between the cognitive performance of MCI patients and theta-gamma coupling. It is still unclear what the distinctions of CFC in AD and MCI are.

The aim of this study was to investigate the CFC properties of rsEEG in AD and MCI patients and HCs. These properties were manifested by examination of the statistical relationships between different EEG oscillations and multiple frequency pairs in the three groups. The investigation included global-level and region-of-interest analysis and exploration of the relationship between the observed couplings and cognitive functional performance.

## 2. Materials and methods

### 2.1. Diagnostic criteria

This study was approved by the Shanghai Clinical Research Ethics Committee and complied with the Declaration of Helsinki. In all cases, written informed consent for research was obtained from the patient or legal guardian. The selected participants were individuals who visited the neurology department at Tongji Hospital in Shanghai between January 2018 and December 2021. The diagnosis of AD was based on the core clinical diagnostic criteria for possible AD dementia set forth in the National Institute on Aging Alzheimer’s Association (NIA-AA) guidelines for Alzheimer’s disease (McKhann et al., 2011), and the “MCI-criteria for the clinical and cognitive syndrome” (Albert et al., 2011) served as the foundation for identifying individuals with MCI. Another inclusion criterion for AD patients included memory decline that had been present for at least 12 months and was accompanied by a trend of progressive deterioration. The criteria for the inclusion of individuals as HC were as follows: (1) no complaints of memory impairment; (2) normal cognitive function; (3) normal performance of activities of daily living; (4) scores in all cognitive areas fell within the normal range or were decreased in only one area; and (5) scores in the normal range on all neuropsychological scales. Participants were excluded if they exhibited any of the following: (1) depression or psychosis; (2) severe primary illness; (3) neurological disorders that can cause cognitive decline (except early AD) (e.g., Parkinson’s disease, brain tumor, epilepsy, encephalitis, hydrocephalus, multiple sclerosis, hepatic encephalopathy, or other disorders); (4) alcohol or drug addiction; and (5) physical disorders such as impaired consciousness or aphasia that made it impossible for the individual to complete the experiment.

All participants were evaluated by neurologists at the Department of Neurology and Memory Clinic at Tongji Hospital in Shanghai according to the specific criteria outlined above and received standardized diagnostic evaluations by specialists, including computed tomography (CT) or magnetic resonance imaging (MRI) scans of the head, blood tests, and comprehensive neuropsychological assessments that included memory assessment, language assessment, executive function assessment, visual space navigation function assessment, the Mini-Mental State Examination (MMSE), the Clinical Dementia Rating scale (CDR), the Instrumental Activities of Daily Living Scale (IADL), the Hachinski Ischemic Score (HIS), the Hamilton Depression Rating Scale (HAMD), and the Hamilton Anxiety Rating Scale (HAMA). The MMSE was used only for screening and not as the sole criterion for identifying individuals with AD and MCI, and HAMD and HAMA scores were used to evaluate the emotional states of the participants. Memory function was measured using the Hopkins Verbal Learning Test-Revised (HVLT-R), which includes the immediate recall, 5-min delayed recall, and 20-min delayed recall tests, and the logical memory test (Wechsler memory scale). Language function was assessed using the Boston Naming Test and the Verbal Fluency Test. Executive function was measured using the Shape Trial Test-A and B (STT-A, STT-B). Visual space navigation function was assessed using the Rey-Osterrieth Complex Figure Test (CFT, including the copy test and the recall test).

### 2.2. Participants

Based on the above criteria, 132 subjects who completed closed-eye resting EEG acquisition were included in this study; the study sample consisted of 43 HCs, 46 MCI patients and 43 AD patients. A part of AD patients were treated with anti-dementia drugs including memantine hydrochloride and donepezil. EEG acquisition was scheduled to be completed within 1 month for the treated patients. Table 1 displays the demographic and clinical characteristics of the participants examined in this study. The delayed recall score was defined as the mean of the scores obtained on the 5-min delayed recall test and the 20-min delayed recall test. The chi-square test was used to compare scores by gender. One-way ANOVA was used to compare age, verbal fluency and immediate recall. Due to the non-normal distribution of the data, education level, scores on the MMSE, HAMA, and HAMD, scores on the delayed recall, logical memory, and Boston naming tests, and scores on both tests of executive function and both tests of visual space navigation function were compared using the Kruskal-Wallis test. The three groups of subjects were matched for gender, age and education level. MCI patients and AD patients had significantly lower MMSE scores, significantly lower memory function scores, and significantly weaker executive and language functions than did HCs.

**TABLE 1:** Demographics of the participants.

	HC (*n* = 43)	MCI (*n* = 46)	AD (*n* = 43)	Between-group differences
Gender (male: female)	25:18	24:22	20:23	χ^2^ = 1.165, *p* = 0.558^a^
Anti-dementia drugs	–	–	14	χ^2^ = 32.400, **p** < **0.001^a^**
Age	70.67 ± 6.10	71.43 ± 7.13	73.79 ± 6.96	*F*(2,129) = 2.529, *p* = 0.086**^b^**
Education (years)	11.33 ± 2.60	10.13 ± 3.32	10.02 ± 3.45	*H* = 5.339, *p* = 0.069**^c^**
MMSE	27.53 ± 1.53	24.28 ± 3.27	18.63 ± 6.56	*H* = 59.707, **p** < **0.001^c^**
HAMA	7.87 ± 4.24	8.00 ± 4.49	7.36 ± 6.20	*H* = 0.793, *p* < 0.673**^c^**
HAMD	5.05 ± 3.66	5.59 ± 4.19	4.84 ± 4.23	*H* = 0.828, *p* = 0.661^c^
**Memory function domain**				
HVLT - Immediate recall	6.70 ± 1.41	4.87 ± 1.56	2.97 ± 1.93	*F*(2,127) = 1.438, **p** < **0.001^b^**
HVLT - Delayed recall	7.14 ± 2.03	2.55 ± 2.58	0.91 ± 1.98	*H* = 75.791, **p** < **0.001^c^**
Logical memory test	8.86 ± 2.49	5.93 ± 2.73	3.34 ± 2.62	*H* = 55.442, **p** < **0.001^c^**
**Language function domain**				
Boston naming test	23.67 ± 3.11	19.70 ± 3.76	15.81 ± 5.70	*H* = 47.926, **p** < **0.001^c^**
Verbal fluency test	14.27 ± 3.16	12.70 ± 3.91	8.41 ± 4.64	*F*(2,127) = 22.409, **p** < **0.001^b^**
**Executive function domain**				
Shape trials test, STT (STT-A)	61.65 ± 19.32	73.76 ± 26.55	111.55 ± 43.22	*H* = 41.589, **p** < **0.001^c^**
Shape trials test, STT (STT-B)	146.67 ± 37.94	192.11 ± 65.79	256.50 ± 58.98	*H* = 45.819, **p** < **0.001^c^**
**Visual space navigation function domain**				
Rey-O complex figure test, ROCF (copy scores)	5.58 ± 0.70	6.28 ± 4.60	8.63 ± 10.31	*H* = 0.67, *p* = 0.715^c^
Rey-O complex figure test, ROCF (recall scores)	4.21 ± 1.49	2.82 ± 2.29	1.29 ± 3.54	*H* = 42.573, **p** < **0.001**^c^

Values are presented as mean ± standard deviation. *P-values* less than 0.05 are shown in bold type. HC, healthy controls; MCI, mild cognitive impairment; AD, Alzheimer’s disease; Anti-dementia drugs include hydrochloride and donepezil; MMSE, Mini-Mental State Examination; HAMA, Hamilton Anxiety Rating Scale, HAMD, Hamilton Depression Rating Scale; HVLT, Hopkins Verbal Learning Test. Higher scores on the STT-A or STT-B test indicate that more time was needed to complete the test and suggest poorer executive performance.

a⁢Chi-square⁢test,Oneb-way⁢analysis⁢of⁢variance,Kruskalc-Wallis⁢test.

### 2.3. EEG collection and preprocessing

Electroencephalography signals were acquired using the NeuroScan 64-conductor SynAmps2 Model 8,050 system (NeuroScan, Charlotte, NC, USA), with electrode caps fixed with 64-conductor Ag/AgCl surface electrodes extended in accordance with the international 10–20 electrode system. The ground electrode was placed between the Fpz and Fz electrodes, and the reference electrode was placed between Cz and Cpz. To reduce interference from the 50 Hz industrial frequency, a trap filter was used to record the closed-eye rsEEG data at a sampling frequency of 1,000 Hz while maintaining the electrode impedance below 10 kΩ. During the experiment, all participants were asked to sit in a chair, remain relaxed, awake and quiet, and wear an EEG cap on their heads while rsEEG data were recorded for 6 min. Manual monitoring prevented the participants from dozing off.

To preprocess the EEG data, the following steps were performed in MATLAB R2017b EEGLAB software (version 14.1.1) (Mathworks, Natick, MA, USA): (1) removal of redundant channels, including the two electrodes placed at the bilateral mastoid and two electrodes of the electrooculogram (EOG); (2) bandpass filtering of the data at 0.5–49 Hz; (3) baseline correction; (4) interpolation of bad channels and visual removal of bad signal segments; (5) removal through independent component analysis (ICA) of artifactual components such as those caused by muscle and eye movements; (6) visual inspection and removal of the remaining EEG segments containing artifacts; and (7) division of the data into 2 s-length non-overlapping data segments and averaging for rereferencing. After preprocessing, it was verified that each participant had at least 30 trials that could be examined.

### 2.4. Cross-frequency coupling analysis

The most frequently researched aspect of CFC is phase amplitude coupling (PAC), and several criteria for assessment of the correlation of its constituent parts currently exist. These include phase locking value (PLV, Mormann et al., 2005), mean vector length (MVL, Canolty et al., 2006), modulation index (MI, Tort et al., 2008), and the general linear model (GLM, Kramer and Eden, 2013). The performance of the four calculation methods listed above was compared by Hülsemann et al. (2019) in the context of regulatory factors such as data length, signal-to-noise ratio, and sampling rate. The results showed that MVL is the most sensitive of the four methods to the modulation of coupling strength and width, and the use of MVL is advised in situations that involve high sampling rates, high signal-to-noise ratios or long data segments. MI was shown to be least affected by confounding factors; therefore, for data with unknown coupling forms and when segments of data are short, MI is recommended.

In this study, the analyses method of data under different conditions are the same. Taking into account that the EEG signal is separated into 2-s data segments, we investigated the PAC mode by computing the MI between the low-frequency phase and the high-frequency amplitude for each electrode. The MI reflects the fact that when a signal experiences phase-amplitude coupling, an inhomogeneous distribution of the high-frequency oscillation amplitude exists that is conditioned by the low-frequency oscillation phase condition. In this study, five conventional EEG bands, namely, delta (1–4 Hz), theta (4–7 Hz), alpha (7–13 Hz), beta (13–30 Hz), and gamma (30–49 Hz), were employed to compute ten distinct coupling characteristics for analysis.

We used f_A_ and f_P_ to denote the high-frequency amplitude and the low-frequency phase frequency ranges to be analyzed, respectively. x (t) Denotes the original EEG signal, and MI was calculated using the following steps:

(1) First, an FIR filter with zero-phase delay was used to filter x(t) within f_A_ and f_P_; the filtered signals are denoted x_f_A__ (t) and x_f_P__ (t). The Hilbert transform was used to obtain the phase time series Φ_f_P__ (t) of x_f_P__ (t) and the amplitude time series A_f_A__ (t) of x_f_A__ (t), thereby constructing a composite time series [Φ_f_P__ (t), A_f_A__ (t)] that represents the amplitude of each phase stage of f_P_. In this way, the amplitude and phase dynamics captured by the envelope A_f_A__ (t) and the instantaneous phase signal Φ_f_P__ (t), respectively, can be processed independently.
(2) Φ_f_P__ (t) was divided into N phase boxes equally, and the average value of the amplitude in each phase box A_f_A__ (t), was calculated. The average value of the amplitude calculated in the jth phase box is expressed as ⟨A_f_A__⟩_Φ_f_P___ (j). We set N to 18, as is done in other literature (Tort et al., 2010).
(3) The relevant entropy measure used to calculate the average amplitude was determined as follows:

$$\text{H}\,\left(p\right)=-\sum_{j=1}^{N}p\,\left(j\right)\,l\,o\,g\,p\,\left(j\right)$$

where p(j) is the normalized amplitude of the jth phase box, i.e.,

$$p\,\left(j\right)=\frac{{\left\langle A_{f_{A}}\right\rangle }_{\Phi_{f_{P}}}\,\left(\text{j}\right)}{\sum_{j=1}^{N}{\left\langle A_{f_{A}}\right\rangle }_{\Phi_{f_{P}}}\,\left(\text{j}\right)}$$

(4) The final entropy-based normalized MI is expressed as.

$$\text{MI}=\frac{{\text{H}}_{\text{max}}-H}{{\text{H}}_{\text{max}}}$$

Thus, the phase amplitude distribution will deviate from the uniform distribution, and the PAC intensity will increase as the MI value increases. If the MI value is 0, there is no PAC. In this study, we first computed the coupling averages for all electrodes at the global level and explored the association between coupling strength at the global level and scores on the clinical scales used to evaluate patients’ performance in several domains of cognitive function. We also compared the coupling means in eight different brain regions: left frontal (FP1, AF3, F1, F3, F5, F7), right frontal (FP2, AF4, F2, F4, F6, F8), left temporal (T7), right temporal (T8), left parietal (CP1, CP3, CP5, TP7, P1, P3, P5), right parietal (CP2, CP4, CP6, TP8, P2, P4, P6), left occipital (PO3, PO5, PO7, O1), and right occipital (PO4, PO6, PO8, O2). This was done to further investigate whether there are variations in coupling strength in various brain regions between the three groups. Furthermore, we examined the correlation between different global-level couplings as well as the correlation between couplings in different regions.

### 2.5. Statistical analysis

A significance threshold of *p* < 0.05 was used in all statistical analyses performed in jamovi software (version 2.2.5) (Sydney, Australia). We first performed a parametric test and a test for normality. Depending on whether the data followed a normal distribution, the Kruskal-Wallis test or analysis of variance (ANOVA) was employed to assess differences among the AD, MCI, and HC groups. If overall significant results were obtained using the Kruskal-Wallis test, separate analyses were performed in which whole brain coupling intensity was compared in each pair of groups using Dwass Steel Crithlow Fligner (DSCF) *post hoc* multiple comparisons. If overall significant results were obtained using the ANOVA test, additional two-by-two comparisons were made using the Turkey method, and the results were corrected using the Bonferroni method. Similarly, to compare the strength of coupling between various frequency bands in the region of interest, we used the Kruskal-Wallis test or ANOVA. Since multiple comparisons of multiple brain regions were conducted simultaneously, false discovery rate (FDR, Benjamini and Yekutieli, 2001) correction was performed on the *p*-values of the test results. *Post hoc* multiple comparisons were performed using DSCF or the Bonferroni method for interband couplings that were still significantly different after correction. In addition, Spearman’s correlation was used to analyze the correlation between CFC properties and clinical cognitive function performance, and the results were corrected by FDR.

## 3. Results

### 3.1. Power analysis

**FIGURE 1:**
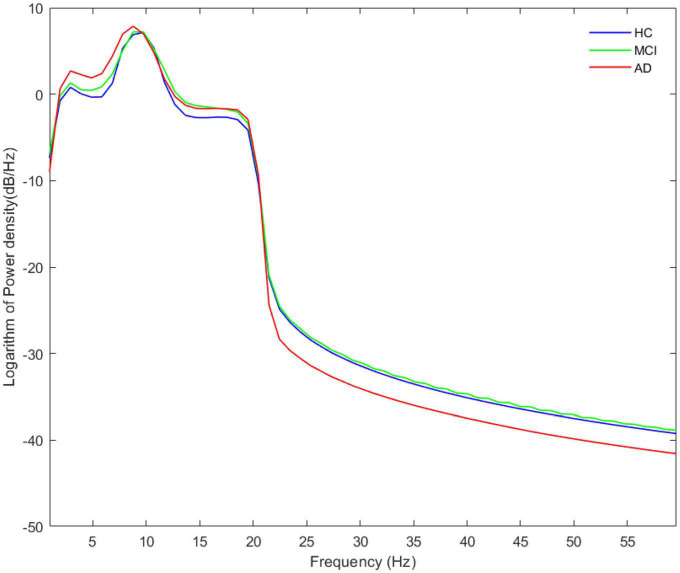
The average absolute EEG power in HCs (blue line), MCIs (green line) and ADs (red line). A legible peak in the alpha band can be seen in all three groups. The *Y*-axis represented the logarithm of power density and the *X*-axis represented frequency bands.

**FIGURE 2:**
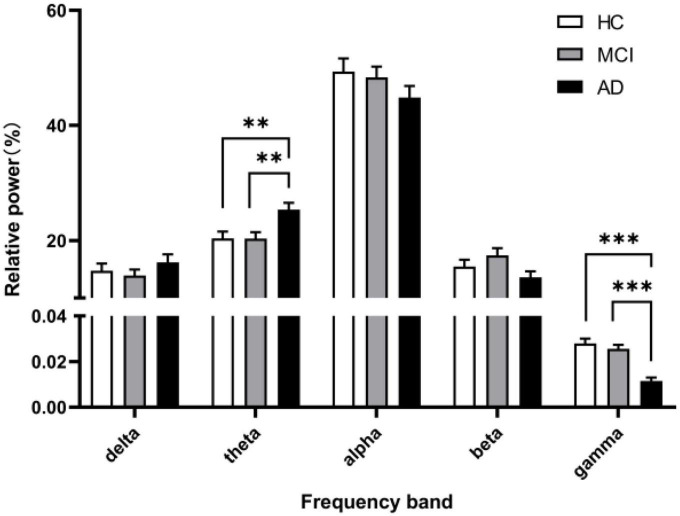
The relative power in five frequency bands. A marked increase of the relative power in theta frequency and a decrease in gamma frequency was observed in the AD group (black columns) compared with the relative power in the HC group (white columns) and MCI group (gray columns). All data were expressed as the mean ± SEM.

As noted by Aru et al. (2015), a clear peak in the power spectrum of the low frequency component is a prerequisite for a meaningful interpretation of any CFC pattern. So before performing the CFC analysis, we calculated the Welch’s power spectral density estimate of the three groups, where the overlap of the data segments was set to 50%. Besides, we calculated and compared the relative power (normalized to the overall power) in five frequency bands. The power spectral density results are shown in Figure 1, and a legible peak in the alpha band can be seen in all three groups. According to statistical analysis of relative power in Figure 2, there were significant differences between the groups in terms of the relative power of the theta and gamma bands. Specifically, the relative power of low-frequency theta oscillations in AD patients was significantly higher than that in HC (*p* = 0.008) and MCI (*p* = 0.006). Also, the relative power of high-frequency gamma oscillations in AD patients was significantly lower than that in HC (*p* < 0.001) and MCI (*p* < 0.001).

### 3.2. Global-level analysis

**FIGURE 3:**
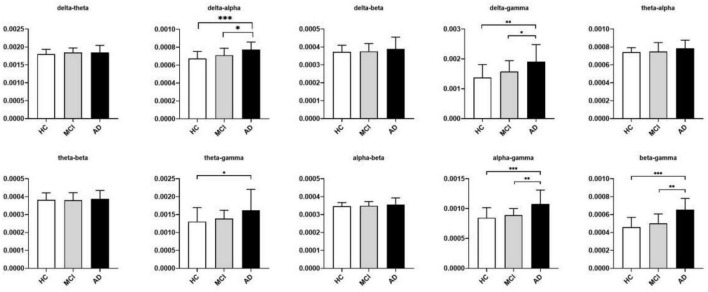
Between-group differences in coupling strength between multiple frequency bands identified using the nonparametric Kruskal-Wallis test with DSCF correction for each class. The histograms show median values and interquartile ranges. The *Y-axis* represented the global average cross-frequency coupling strength. Relative to the HCs, AD patients showed higher levels of delta-alpha, delta-gamma, theta-gamma, alpha-gamma, and beta-gamma coupling strengths. Relative to the MCI patients, AD patients showed higher levels of delta-alpha, delta-gamma, alpha-gamma, and beta-gamma coupling strengths. *p < 0.05, **p < 0.01, and ***p < 0.001. See Table 2 for more detailed statistical information. HC, healthy controls; MCI, mild cognitive impairment; AD, Alzheimer’s disease.

**FIGURE 4:**
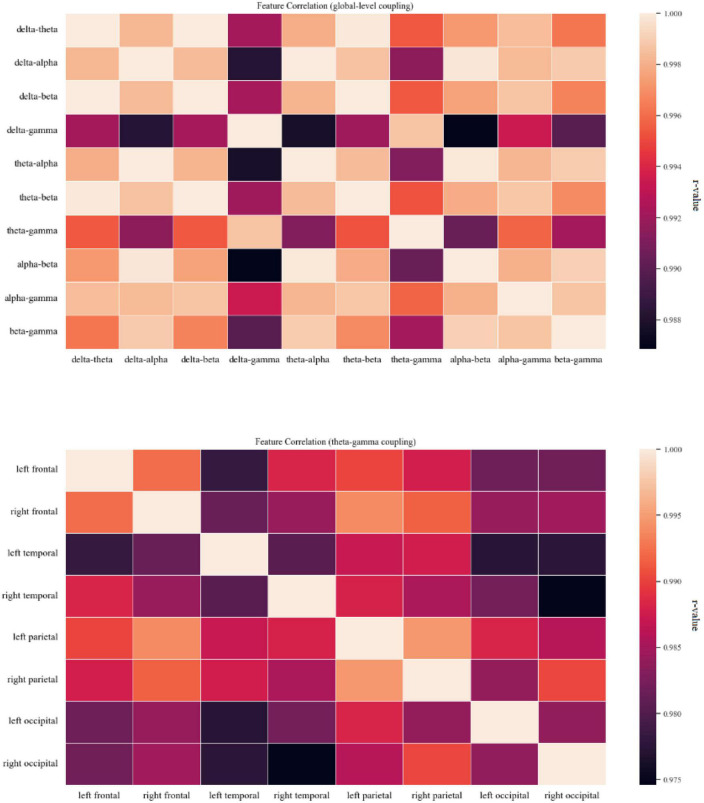
Coupling feature correlation. The panel on the top shows a thermal correlation diagram for global-level coupling, and the panel on the bottom shows a thermal correlation diagram of theta-gamma coupling between brain regions. Lighter colors indicate stronger correlation. High correlations (*r* ≥ 0.75) between coupling features were found.

The significant differences between groups at each coupling intensity that remained after the non-parametric Kruskal-Wallis test for the global average coupling between multiple frequency bands are shown in Figure 3 and Table 2. The delta-alpha (*p* < 0.001), delta-gamma (*p* = 0.003), theta-gamma (*p* = 0.024), alpha-gamma (*p* < 0.001), and beta-gamma (*p* < 0.001) coupling intensities were substantially greater in the AD patients than in the HCs. Relative to the MCI patients, the AD patients showed significantly higher levels of delta-alpha (*p* = 0.030), delta-gamma (*p* = 0.025), alpha-gamma (*p* = 0.002), and beta-gamma (*p* = 0.001) coupling strength. Furthermore, there was no discernible difference in the strength of the global-level coupling in the HC and MCI groups. The correlations between various couplings are displayed in Figure 4; strong correlations were found.

**TABLE 2:** Results of global coupling analysis among multiple frequency bands in healthy controls, individuals with mild cognitive impairment, and individuals with Alzheimer’s disease.

	Kruskal–Wallis	Between-group differences		
		**HC-MCI**	**MCI-AD**	**HC-AD**
Delta-theta	*H* = 1.665, *p* = 0.435	0.851	0.466	0.628
Delta-alpha	*H* = 15.203, *p* < 0.001	0.326	**<0.001**	**0.030**
Delta-beta	*H* = 4.211, *p* = 0.122	0.726	0.119	0.356
Delta-gamma	*H* = 12.739, *p* = 0.002	0.479	**0.003**	**0.025**
Theta-alpha	*H* = 5.403, *p* = 0.067	0.760	0.050	0.302
Theta-beta	*H* = 0.644, *p* = 0.725	0.931	0.741	0.847
Theta-gamma	*H* = 8.452, *p* = 0.015	0.706	**0.024**	0.055
Alpha-beta	*H* = 2.589, *p* = 0.274	0.915	0.268	0.479
Alpha-gamma	*H* = 21.404, *p* < 0.001	0.392	**<0.001**	**<0.001**
Beta-gamma	*H* = 24.085, *p* < 0.001	0.440	**<0.001**	**<0.001**

The *post hoc P*-values have been DSCF-corrected for multiple comparisons between groups. *P*-values less than 0.05 are presented in bold type. HC, healthy controls; MCI, mild cognitive impairment; AD, Alzheimer’s disease.

### 3.3. Region-of-interest analysis

**FIGURE 5:**
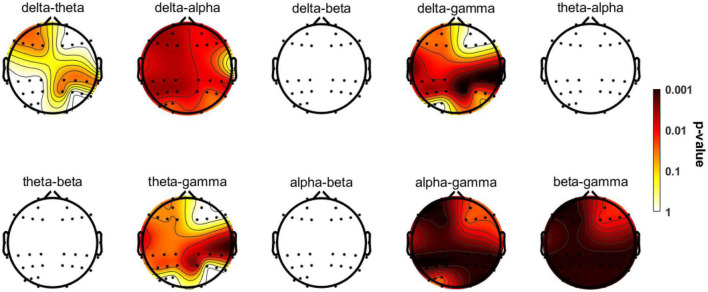
Topographic distributions of statistically significant differences in CFC among the three groups. The values are color coded. Redder color indicates smaller *P*-value. Statistically significant differences occurred in delta-theta, delta-alpha, and delta/theta/alpha/beta-gamma couplings. See Table 3 for more detailed *post hoc* statistical information.

The significant group differences identified using the Kruskal-Wallis test with FDR correction for each region of interest are shown in Figure 5. The three groups did not display statistically significant differences in delta-beta, theta-alpha, theta-beta, or alpha-beta coupling, but they differed substantially in all other couplings among multiple brain regions. The post-event comparison results are shown in Table 3. Notably, differences between HCs and AD patients are widespread across multiple couplings and multiple brain regions, while only delta-gamma coupling in the right temporal lobe (*p* = 0.022) and the parietal lobe (*p* = 0.005) and theta-gamma coupling in the right temporal lobe (*p* = 0.049) and the parietal lobe (*p* = 0.029) showed significant variations between HCs and MCI patients. More interestingly, only theta-gamma coupling strength in the right temporal lobe showed between-group differences; it was stronger in MCI patients than in HCs and stronger in AD patients than in individuals with MCI.

**TABLE 3:** Results of coupling analysis among multiple region-of-interest and multiple frequency bands between healthy controls, mild cognitive impairment and Alzheimer’s disease.

		Krusal–Wails	Between-group differences				Krusal–Wails	Between-group differences		
			HC-MCI	MCI-AD	HC-AD			HC-MCI	MCI-AD	HC-AD
Delta-theta	Left frontal	*H* = 9.239, *p* = 0.040	–	0.006	–	Right frontal	*H* = 0.665, *p* = 0.819	–	–	–
	Left temporal	*H* = 1.269, *p* = 0.787	–	–	–	Right temporal	*H* = 2.255, *p* = 0.787	–	–	–
	Left parietal	*H* = 1.055, *p* = 0.787	–	–	–	Right parietal	*H* = 10.203, *p* = 0.040	–	–	0.007
	Left occipital	*H* = 0.366, *p* = 0.833	–	–	–	Right occipital	*H* = 1.373, *p* = 0.787	–	–	–
Delta-alpha	Left frontal	*H* = 14.567, *p* = 0.004	–	0.034	0.001	Right frontal	*H* = 10.546, *p* = 0.010	–	0.042	0.006
	Left temporal	*H* = 10.241, *p* = 0.010	–	–	0.007	Right temporal	*H* = 5.432, *p* = 0.066	–	–	–
	Left parietal	*H* = 13.922, *p* = 0.004	–	–	<0.001	Right parietal	*H* = 11.581, *p* = 0.008	–	–	0.002
	Left occipital	*H* = 8.035, *p* = 0.023	–	0.034	0.042	Right occipital	*H* = 7.821, *p* = 0.023	–	–	0.014
Delta-beta	Left frontal	*H* = 2.661, *p* = 0.422	–	–	–	Right frontal	*H* = 3.904, *p* = 0.284	–	–	–
	Left temporal	*H* = 0.988, *p* = 0.610	–	–	–	Right temporal	*H* = 1.455, *p* = 0.552	–	–	–
	Left parietal	*H* = 4.091, *p* = 0.284	–	–	–	Right parietal	*H* = 4.894, *p* = 0.284	–	–	–
	Left occipital	*H* = 4.099, *p* = 0.284	–	–	–	Right occipital	*H* = 1.643, *p* = 0.552	–	–	–
Delta-gamma	Left frontal	*H* = 7.507, *p* = 0.031	–	–	0.030	Right frontal	*H* = 5.927, *p* = 0.059	–	–	–
	Left temporal	*H* = 19.193, *p* < 0.001	–	0.005	<0.001	Right temporal	*H* = 19.029, *p* < 0.001	**0.022**	–	<0.001
	Left parietal	*H* = 11.647, *p* = 0.006	–	0.023	0.004	Right parietal	*H* = 18.077, *p* < 0.001	**0.005**	–	<0.001
	Left occipital	*H* = 5.484, *p* = 0.064	–	–	–	Right occipital	*H* = 7.691, *p* = 0.031	–	–	0.019
Theta-alpha	Left frontal	*H* = 10.009, *p* = 0.197	–	–	–	Right frontal	*H* = 9.345, *p* = 0.224	–	–	–
	Left temporal	*H* = 1.120, *p* = 0.467	–	–	–	Right temporal	*H* = 3.100, *p* = 0.224	–	–	–
	Left parietal	*H* = 2.793, *p* = 0.197	–	–	–	Right parietal	*H* = 6.836, *p* = 0.128	–	–	–
	Left occipital	*H* = 3.558, *p* = 0.224	–	–	–	Right occipital	*H* = 3.405, *p* = 0.224	–	–	–
Theta-beta	Left frontal	*H* = 0.198, *p* = 0.906	–	–	–	Right frontal	*H* = 1.130, *p* = 0.855	–	–	–
	Left temporal	*H* = 1.688, *p* = 0.855	–	–	–	Right temporal	*H* = 0.710, *p* = 0.855	–	–	–
	Left parietal	*H* = 1.505, *p* = 0.855	–	–	–	Right parietal	*H* = 3.524, *p* = 0.855	–	–	–
	Left occipital	*H* = 1.856, *p* = 0.855	–	–	–	Right occipital	*H* = 0.581, *p* = 0.855	–	–	–
Theta-gamma	Left frontal	*H* = 7.143, *p* = 0.045	–	–	0.030	Right frontal	*H* = 2.146, *p* = 0.342	–	–	–
	Left temporal	*H* = 13.336, *p* = 0.004	–	0.038	0.002	Right temporal	*H* = 19.001, *p* < 0.001	**0.049**	0.038	<0.001
	Left parietal	*H* = 8.601, *p* = 0.028	–	0.034	0.026	Right parietal	*H* = 12.258, *p* = 0.005	**0.029**	–	0.004
	Left occipital	*H* = 3.942, *p* = 0.159	–	–	–	Right occipital	*H* = 5.630, *p* = 0.080	–	–	–
Alpha-beta	Left frontal	*H* = 1.617, *p* = 0.560	–	–	–	Right frontal	*H* = 2.880, *p* = 0.560	–	–	–
	Left temporal	*H* = 1.159, *p* = 0.560	–	–	–	Right parietal	*H* = 3.432, *p* = 0.560	–	–	–
	Left parietal	*H* = 4.794, *p* = 0.560	–	–	–	Right parietal	*H* = 3.432, *p* = 0.560	–	–	–
	Left occipital	*H* = 1.380, *p* = 0.560	–	–	–	Right occipital	*H* = 2.545, *p* = 0.560	–	–	–
Alpha-gamma	Left frontal	*H* = 16.709, *p* < 0.001	–	0.024	<0.001	Right frontal	*H* = 7.873, *p* = 0.022	–	–	0.028
	Left temporal	*H* = 11.723, *p* = 0.005	–	0.020	0.005	Right temporal	*H* = 9.514, *p* = 0.012	–	–	0.006
	Left parietal	*H* = 16.325, *p* < 0.001	–	0.014	<0.001	Right parietal	*H* = 17.449, *p* < 0.001	–	0.026	<0.001
	Left occipital	*H* = 7.620, *p* = 0.022	–	0.039	–	Right occipital	*H* = 15.520, *p* < 0.001	–	–	<0.001
Beta-gamma	Left frontal	*H* = 13.112, *p* = 0.001	–	0.007	0.005	Right frontal	*H* = 8.856, *p* = 0.012	–	–	0.016
	Left temporal	*H* = 12.234, *p* = 0.002	–	0.045	0.002	Right temporal	*H* = 14.258, *p* < 0.001	–	0.006	0.002
	Left parietal	*H* = 19.117, *p* < 0.001	–	0.009	<0.001	Right parietal	*H* = 20.599, *p* < 0.001	–	0.016	<0.001
	Left occipital	*H* = 24.418, *p* < 0.001	–	<0.001	<0.001	Right occipital	*H* = 13.880, *p* < 0.001	–	–	<0.001

*P*-values are FDR-corrected for Kruskal–Wallis test. The *post hoc P*-values are DSCF-corrected for multiple comparisons between groups and only the significant parts are shown in the figure. Significant *p*-values between HC and MCI are in bold. HC, healthy control; MCI, mild cognitive impairment; AD, Alzheimer’s disease.

The theta-gamma couplings in eight brain regions were selected as representatives and used to examine the correlations among couplings of different regions of interest. Figure 5 shows that the correlations between the coupling results for different regions of interest were strong.

### 3.4. Clinical relevance

**FIGURE 6:**
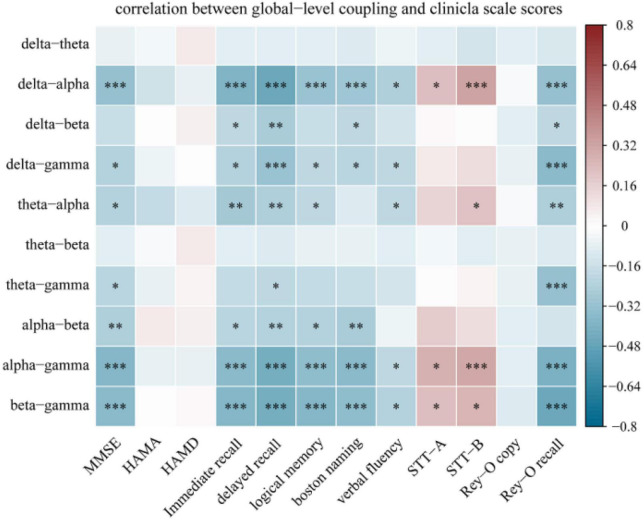
Spearman correlation analysis between global-level coupling and clinical scale scores. The *Y*-axis represents the global-level coupling. Red indicates a positive correlation, and blue indicates a negative correlation. The darker the color, the larger the correlation coefficient. Correlations between MMSE score and memory function accounted for the largest proportion (26.42%) of significant correlations. *p < 0.05, **p < 0.01, and ***p < 0.001. Memory function was measured using the Hopkins Verbal Learning Test-Revised (HVLT-R), which includes tests of both immediate recall and delayed recall, and the logical memory test (Wechsler memory scale). Language function was assessed using the Boston Naming Test and the Verbal Fluency Test. Executive function was measured using the Shape Trial Test-A and B (STT-A and STT-B). Visual space navigation function was assessed using the Rey-Osterrieth Complex Figure Test (CFT), which includes a copy test and a recall test.

Figure 6 displays the results of Spearman correlation analysis of the relationship between global-level coupling and clinical scores; see Supplementary Table 1 for detailed statistical results. There were significant weak negative correlations between MMSE scores and coupling strengths. In addition, scores on the immediate memory and delayed memory tests, which assess verbal memory function, the Boston naming test and the verbal fluency test, which assess language function, and the complex figure recall test, which assesses visual spatial memory, all displayed significant negative weak correlations with multiple couplings. The trial-making test scale scores for evaluating executive function were mainly related to delta-alpha (*r* = 0.225, *p* = 0.037; *r* = 0.329, *p* = 0.001), theta-alpha (*r* = 0.223, *p* = 0.043), alpha-gamma (*r* = 0.276, *p* = 0.020; *r* = 0.306, *p* = 0.001), and beta-gamma (*r* = 0.233, *p* = 0.037; *r* = 0.265, *p* = 0.013) couplings; for these couplings, the correlation was weakly positive. Furthermore, we calculated the percentage of significant correlations that occurred in different cognitive domains. Correlations between MMSE score and memory function accounted for the largest proportion (26.42%) of significant correlations.

**FIGURE 7:**
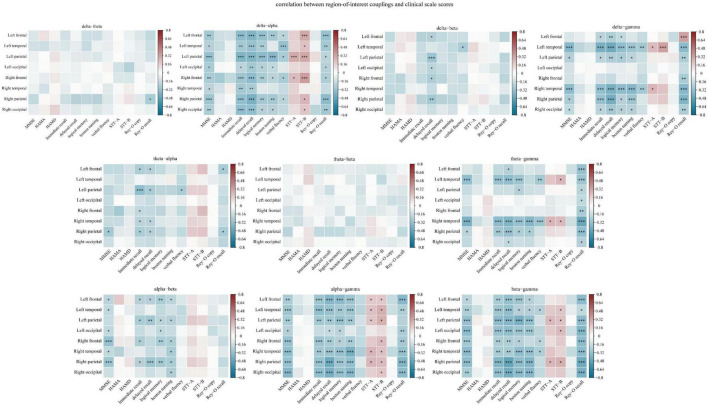
Spearman correlation analysis of the relationship between region-of-interest couplings and clinical scale scores. The *Y*-axis represents the region-of-interest couplings. Red indicates a positive correlation, and blue indicates a negative correlation. The darker the color, the larger the correlation coefficient. The memory function domain accounted for the largest proportion (29.68%) of the significant correlations. *p < 0.05, **p < 0.01, and ***p < 0.001. Memory function was measured using the Hopkins Verbal Learning Test-Revised (HVLT-R), which includes tests of both immediate recall and delayed recall, and the logical memory test (Wechsler memory scale). Language function was assessed using the Boston Naming Test and the Verbal Fluency Test. Executive function was measured using the Shape Trial Test-A and B (STT-A and STT-B). Visual space navigation function was assessed using the Rey-Osterrieth Complex Figure Test (CFT), which includes a copy test and a recall test.

The correlations between coupling in each region of interest and cognitive scale scores are shown in Figure 7, and the detailed results are shown in Supplementary Table 2 in the Supplementary material. With the exception of theta-beta coupling, coupling in various regions of interest exhibited significant correlation with clinical scale scores. Among the cognitive domains, the memory function domain accounted for the largest proportion (29.68%) of the significant correlations.

## 4. Discussion

To explore alterations in neural mechanisms that occur at different stages of cognitive dysfunction, we analyzed changes in the power of the spontaneous EEG activities and changes in the intensity of multiple CFCs of rsEEG in AD and MCI patients and HCs. In the case of global-level coupling, the intensities of delta-alpha, delta-gamma, theta-gamma, alpha-gamma, and beta-gamma coupling varied significantly among the three groups. In regard to coupling in specific regions of interest, the single most striking observation to emerge from the data comparison was that theta-gamma coupling in the right temporal lobe tended to be stronger in individuals with MCI than in HCs and stronger still in AD patients.

### 4.1. Increased theta power and decreased gamma power

By analyzing the Welch’s power spectrum of rsEEG, we found increased theta power and decreased gamma power in AD patients, which were consistent with the findings in some previous reports (Ishii et al., 2017; Jafari et al., 2020). Notably, theta band activity has been suggested to play an apparent role in episodic memory regulation and correlate with AD pathology by numerous studies in mouse AD models and humans [for detailed review, see Colgin (2013), Jafari et al. (2020), Mehak et al. (2022)]. And in cognitively normal elderly subjects, cerebrospinal fluid (CSF) total-tau and phosphorylated tau (*p*-tau) levels as well as the combined p-tau/ratio are associated with relative EEG theta power (Ishii et al., 2017). Therefore, the increase of theta rhythm in our study may be an early phenomenon of neurodegeneration that reflects memory deficits of AD patients. Moreover, gamma oscillations are linked to higher-order cognitive functions including information processing (Fries et al., 2008; Leicht et al., 2021), perception (Melloni et al., 2007; Naito et al., 2022), attention (Jensen et al., 2007; Magazzini and Singh, 2018), and memory (Kucewicz et al., 2017). A study of more than 300 individuals by Gaubert et al. (2019) explored the effects of AD neurodegeneration and amyloid- deposition on EEG metrics, they found a non-linear relationship between amyloid burden and EEG metrics, following a U-shape curve for gamma power, where patients’ gamma power decreased as amyloid load exceeded a certain threshold. Based on these findings, the abnormal gamma oscillations power in AD patients and the absence of abnormal power in MCI patients observed in this study may reflect a possible compensatory mechanism early in the onset of AD, which can be overwhelmed by a higher amyloid load during continued deterioration of the disease, ultimately leading to multiple cognitive abnormalities in patients. Since no relevant pathological tests were performed, further studies are needed to support this hypothesis.

### 4.2. Enhanced global-level CFC in AD patients

According to our results, AD patients displayed stronger coupling between delta and alpha, delta and gamma, theta and gamma, alpha and gamma, and beta and gamma rhythms than did HCs, a finding that is partially consistent with the results reported by Wang et al. (2017). AD patients showed higher levels of coupling between delta and alpha, delta and gamma, alpha and gamma, and beta and gamma than did MCI patients. No significant increase was found in MCI patients compared with HCs. A recent review (Yakubov et al., 2022) concluded that the results of brain CFC reveal a trend that links a significant increase in CFC to increased demand for cognitive processing and suggested that enhanced coupling may result from such a demand. When measurable changes in the brain such as abnormal levels of beta-amyloid, changes in tau protein, and other changes related to the occurrence of AD occur, the brain compensates to allow individuals to continue functioning normally (Alzheimer’s Association, 2022). Based on this, we think that the enhanced global coupling observed in AD patients may indicate a need to expend more neural resources during cognitive processing to blunt the effects of changes caused by AD.

Coupling between delta and gamma rhythms has been found to increase in anxiogenic situations (Knyazev et al., 2005). In our study, enhanced delta-gamma coupling was observed, but HAMA and HAMD scores did not differ among the HC, MCI and AD groups. This may indicate that coupling alterations are more sensitive than neuropsychological assessment in detecting anxiogenic situations or that changes in delta-gamma coupling may also be related to causes other than anxiety. In general, more research is needed to support our interpretation of the cause of the observed alterations in delta-gamma coupling. The coupling between theta/alpha and gamma rhythms is thought to be functionally relevant to the maintenance of working memory. In a review, Roux and Uhlhaas (2014) concluded that maintenance of sensory-spatial working memory involves alpha-gamma coupling, whereas maintenance of sequential working memory information relies on theta-gamma coupling. Therefore, the increased theta-gamma and alpha-gamma couplings observed in the AD patients in our study may be related to their decreased working memory.

We note that in comparative studies of global-level CFC in HCs, AD patients and MCI patients, inconsistent results have been obtained. For example, patients with MCI who progressed to AD were found to have significantly lower global theta-gamma coupling than patients with MCI who remained stable (Musaeus et al., 2020). However, enhanced global-level coupling was found in our study and in the study by Wang et al. (2017). The conflicting results suggest that further investigation is necessary to determine exactly how global-level CFC varies with the severity of neurocognitive impairment.

### 4.3. Enhanced region-of-interest CFC in AD patients and MCI patients

Analysis of the region of interest can help us further understand which region coupling features best characterize the differences among the three groups. Significant differences among the three groups were found in multiple brain regions with multiple couplings. The post-event between-group comparison results showed that most of the significant differences occurred between HCs and AD patients, followed by those between MCI patients and AD patients. This may indicate that enhanced coupling is more obvious at later stages of the disease.

Regarding the differences between HCs and MCI patients, delta-gamma coupling in the right parietal lobe and the right temporal lobe and theta-gamma coupling in the right parietal lobe were significantly higher in MCI patients than in HCs but were not significantly different between MCI patients and AD patients. We speculate that these three coupling modes are altered in early AD and then remain stable until late stages of the disease. Clinically, early-stage AD is typically characterized by memory disturbances that result from neurodegeneration in the medial temporal lobe. As the disease progresses, this neurodegeneration gradually spreads to the temporal and parietal cortices and, eventually, to most of the cortex (Yu et al., 2021). This may explain the reported differences in delta-gamma coupling and theta-gamma coupling differences between HCs, MCI patients and AD patients. The spread of AD pathology may lead to a region-specific increase in coupling between delta/theta and gamma.

Interestingly, we discovered that only theta-gamma coupling in the right temporal lobe displayed a tendency in which MCI was stronger than HC and that it was stronger in AD patients than in MCI patients, while global-level coupling intensity did not reflect this pattern of change. We speculate that the theta-gamma coupling changes we observed in the temporal lobe may increase over time as the illness progresses and that these changes might provide a useful marker of the disease process. However, since no relevant pathological tests were performed, it could not be proven that the deficits observed in the MCI patients included in this study were caused by AD etiology; further studies are needed to support this hypothesis.

### 4.4. Correlation between CFC features and clinical characteristics

Various cognitive processes may be related to coupling between different frequency bands. Regarding the relevance of the relationship between global-level coupling and clinical scales, we found substantial weak negative correlations between multiple couplings and MMSE scores, memory cognitive domain scale scores, and language function scale scores as well as significant weak positive correlations between delta-alpha, alpha-gamma, and beta-gamma couplings and scores on tests of executive function. Musaeus et al. (2020) found global theta-gamma coupling to be correlated with score on Addenbrooke’s Cognitive Examination (ACE), but Wang et al. (2017) did not observe a marked link between neurocognitive performance and the theta-gamma coupling intensity of rsEEG. The differences in the reported results may be due to differences in sample size and in the methods used to assess coupling.

Regarding the relevance of the relationship between coupling in regions of interest and clinical scales, only theta-beta coupling failed to show a significant association with clinical scale scores. A substantial positive association between language test results and theta-gamma coupling of the rsEEG in the posterior cingulate cortex was discovered by Vanneste et al. (2021); conversely, we found a significant weak negative correlation between these two measures. The heterogeneity of the results may be due to variability in the coupling assessment techniques used; Vanneste et al. (2021) used power-power CFC, while we calculated the phase-amplitude CFC.

Indeed, coupling in different brain regions has been found to be widely associated with multiple domains of cognitive function. We found that CFC that showed significant correlations with the memory function domain accounted for the largest proportion of significant correlations. Studies have been performed to explore the relationship between CFC and memory in AD and MCI. Theta-gamma coupling has emerged as the most potent predictor of 1-back and 2-back verbal working memory performance in AD and MCI (Goodman et al., 2018), and a significant association between theta-gamma coupling during the 2-back working memory task and performance on other cognitive tasks that require ordering in MCI patients was also found (Brooks et al., 2020). However, these studies only discussed the relationship between memory and theta-gamma coupling since theta-gamma coupling has been found to underlie working memory processes (Sauseng et al., 2008; Roux and Uhlhaas, 2014; Goodman et al., 2018; Yakubov et al., 2022). However, a role for other couplings in memory has also been reported in recent years (Daume et al., 2017; Liang et al., 2021). In our study, not only was theta-gamma coupling found to be significantly correlated with memory function scores, but multiple couplings in multiple regions were also found to be significantly associated with memory function domains. This suggests that future task-state studies of the relationship between memory and CFC in MCI or AD should take into account couplings between other frequency bands.

Furthermore, some research have already looked at the link between non-invasive brain modulation and CFC in healthy persons or psychiatric disorders. Bahramisharif et al. (2015) found that basal ganglia deep brain stimulation (DBS) on cross-frequency neuronal interactions between the broadband gamma amplitude and phase of beta and low gamma bands can impact not only the motor cortex of Parkinson’s disease patients, but also the visual cortex of obsessive-compulsive disorder patients. Nevertheless, to date, just a few mice research have explored the relationship between non-invasive brain stimulation and CFC in AD or MCI. For example, Etter et al. (2019) discovered that spatial memory deficits, hippocampal slow gamma oscillations and theta-gamma CFC of J20-amyloid precursor protein (J20-APP) AD mouse model can be restored after optogenetic stimulation. Besides, cross-frequency transcranial alternating current stimulation (CF-tACS), which can be used to deliver customizable waveforms that mimic endogenous phase-amplitude coupling activity patterns, has been explored in several areas, including working memory (Alekseichuk et al., 2016; Abubaker et al., 2021), verbal-long term memory (Lara et al., 2018), cognitive control (Riddle et al., 2021), and so on. By applying CF-tACS to externally modulated the interaction of theta and gamma rhythms, Alekseichuk et al. (2016) demonstrated the role of theta-gamma CFC in human prefrontal cortex for working memory, where enhancement of working memory performance and increase of global neocortical connectivity were observed when bursts of high gamma oscillations coincided with the peaks of the theta waves. These findings suggest that CFCs might serve as targeted markers for non-invasive brain modulation, and that CFC brain stimulations may be a promising tool for modulating daily cognitive performance and treating some certain neuropsychiatric disorders. However, further research is needed to validate it before it can be utilized in clinical diagnosis and therapy.

## 5. Conclusion

In this study, the power spectrum and CFC between different frequency bands of rsEEG was investigated in 43 healthy controls, 46 MCI patients and 43 AD patients. Increased theta power and decreased gamma power were found in AD patients. Moreover, the results showed that global coupling between delta and alpha bands and global coupling between gamma and low-frequency bands were substantially enhanced in AD patients compared with HCs and MCI patients, while delta-gamma coupling and theta-gamma coupling in the right temporal and parietal lobes were significantly enhanced in MCI patients compared with HCs. Most importantly, theta-gamma coupling in the right temporal lobe showed between-group differences in which it was stronger in MCI patients than in HCs and stronger in AD patients than in MCI patients. These differences may be a useful marker of the disease process. Furthermore, multiple CFC properties were found to correlate significantly with specific cognitive domains, especially the memory function domain.

We also examined the correlations between various couplings and the correlations between the couplings of different brain regions. High correlations between coupling features were found in this study; we believe that this suggests that the distinction between coupling between different frequency bands may be low. This could be related to the fact that the concrete patterns represented by the various couplings themselves are still not entirely clear. Therefore, more research is needed to characterize the specific differences between coupling features in the future. High correlations between the theta-gamma couplings of different regions of interest were also found, indicating that the similarity of the coupling mechanisms found in different regions may be high. Additional research should be conducted to investigate this, as we only investigated theta-gamma coupling in our analysis. Overall, although the CFC findings are pertinent to a range of clinical features, cognitive assessments, and task performance, a more distinct pattern has not yet been discernible throughout the entire study project.

Our comprehension of the pathogenic pathways in our patients’ cases may be improved by these preliminary findings, which offer fresh evidence regarding the mechanisms that underlie brain activity disorders in AD and MCI. Additionally, we also conducted a study to investigate the differences in cognitive and coupling characteristics of the medication group compared to the non-medication group in the AD patients. Our findings revealed no significant difference in cognitive tests between the two groups. However, in terms of coupling characteristics, the global delta-gamma coupling of the medication group was significantly lower than that of the non-medication group. Additionally, we observed various coupling differences in multiple brain regions of the temporal lobe, parietal lobe, and frontal lobe in the local coupling analysis. It is important to note that these differences cannot be solely attributed to drug intake. In order to clarify the relationship between drugs and coupling, and to verify the role of coupling indicators in the course of the disease, future analysis should focus on the changes in coupling characteristics before and after medication. Due to lack of data of duration of disease and no difference in the sex of the participants was found in this study, we do not know whether sex or duration of disease will have an impact on CFC, and future research can take these two factors into account. And since no classification was performed, we do not know whether the coupling features described here effectively distinguish among different groups of subjects; this point can be addressed in future work. Moreover, previous research has shown that CFC also occurs across different brain regions (Vanneste et al., 2021). This suggests that future studies should further investigate this type of coupling since our study only measured CFC in the same brain areas. In addition, the directionality of coupling has been discussed by several academics (Jiang et al., 2015), and this also requires further investigation in a follow-up study. In terms of sample size, our study is a small-scale clinical trial, and future research should increase the sample size as much as feasible to confirm the findings. Finally, further research in combination with non-invasive brain stimulation is needed to validate the causal relationship between CFC and manifestations of AD or MCI disease before it can be utilized in clinical diagnosis and therapy in the future.

## Data availability statement

The datasets analyzed during the current study are not publicly available due to privacy but are available from the corresponding authors on reasonable request. Requests to access the datasets should be directed to liyj@i.shu.edu.cn and doctorLiyunxia@163.com.

## Ethics statement

The studies involving human participants were reviewed and approved by the Shanghai Clinical Research Ethics Committee. The patients/participants provided their written informed consent to participate in this study.

## Author contributions

XC: conceptualization, methodology, software, visualization, and writing-original draft. YiL: supervision and writing-review and editing. WZ: resources and data curation. RL: project administration and resources. XY: resources and software. YuL: funding acquisition and project administration. ML: writing-review and editing and formal analysis. All authors contributed to the article and approved the submitted version.
