# Supplementary material for: Multiple cross-frequency coupling analysis of resting-state EEG in patients with mild cognitive impairment and Alzheimer’s disease
Source: Front Aging Neurosci. 2023 Aug 4;15:1142085. doi: 10.3389/fnagi.2023.1142085 (PMC10436577; doi:10.3389/fnagi.2023.1142085)
Supplement: Supplementary file 1 [file Data_Sheet_1.docx]

Supplementary Material

Multiple cross-frequency coupling analysis of resting-state EEG in patients with mild cognitive impairment and Alzheimer's disease

Xi Chen^1^, Yingjie Li^1*^, Wei Zhang^2^, Renren Li^2^, Xiao Yuan^2^, Yunxia Li^2**^

^1^School of Communication and Information Engineering, Shanghai Institute for Advanced Communication and Data Science, Shanghai University, Shanghai 200444, China

^2^Department of Neurology, Tongji Hospital, School of Medicine, Tongji University, Shanghai 200092, China

*** Correspondence:** Yingjie Li: liyj@i.shu.edu.cn (Y.L.);

****co-correspondence:** Yunxia Li: doctorLiyunxia@163.com (Y.L.);

# Supplementary Tables

**Supplementary Table 1**. Correlations between cognitive scale scores and global-level couplings.

|  |  | MMSE | HAMA | HAMD | Memory function | | | Language function | | Executive function | | Visual space navigation function | |
| --- | --- | --- | --- | --- | --- | --- | --- | --- | --- | --- | --- | --- | --- |
|  |  |  |  |  | HVLT-  Immediate recall | HVLT-  Delayed recall | Logical memory test | Boston naming test | Verbal fluency test | STT-A | STT-B | ROCF  (copy scores) | ROCF  (recall scores) |
| delta-theta | Spearman's rho | -0.073 | -0.034 | 0.065 | -0.093 | -0.082 | -0.089 | -0.103 | -0.060 | -0.085 | -0.135 | -0.086 | -0.122 |
|  | p-value | 0.400 | 0.952 | 0.740 | 0.323 | 0.352 | 0.349 | 0.270 | 0.501 | 0.572 | 0.300 | 0.534 | 0.186 |
| delta-alpha | Spearman's rho | -0.322 | -0.158 | -0.077 | -0.396 | -0.450 | -0.308 | -0.293 | -0.255 | 0.225 | 0.329 | -0.030 | -0.322 |
|  | p-value | **<0.001** | 0.640 | 0.740 | **<0.001** | **<0.001** | **<0.001** | **<0.001** | **0.030** | **0.037** | **<0.001** | 0.812 | **<0.001** |
| delta-beta | Spearman's rho | -0.172 | 0.011 | 0.063 | -0.196 | -0.265 | -0.175 | -0.197 | -0.134 | 0.021 | 0.015 | -0.088 | -0.204 |
|  | p-value | 0.061 | 0.952 | 0.740 | **0.036** | **0.004** | 0.059 | **0.042** | 0.183 | 0.910 | 0.878 | 0.534 | **0.030** |
| delta-gamma | Spearman's rho | -0.236 | -0.060 | -0.006 | -0.235 | -0.318 | -0.203 | -0.220 | -0.198 | 0.070 | 0.113 | -0.070 | -0.358 |
|  | p-value | **0.012** | 0.942 | 0.944 | **0.014** | **<0.001** | **0.042** | **0.024** | **0.048** | 0.619 | 0.327 | 0.534 | **<0.001** |
| theta-alpha | Spearman's rho | -0.234 | -0.181 | -0.112 | -0.273 | -0.254 | -0.196 | -0.107 | -0.205 | 0.144 | 0.223 | -0.019 | -0.244 |
|  | p-value | **0.012** | 0.640 | 0.740 | **0.005** | **0.005** | **0.043** | 0.270 | **0.048** | 0.216 | **0.043** | 0.829 | **0.008** |
| theta-beta | Spearman's rho | -0.081 | 0.028 | 0.075 | -0.082 | -0.106 | -0.073 | -0.069 | -0.096 | -0.048 | -0.082 | -0.077 | -0.111 |
|  | p-value | 0.392 | 0.952 | 0.740 | 0.354 | 0.254 | 0.411 | 0.432 | 0.350 | 0.739 | 0.478 | 0.534 | 0.210 |
| theta-gamma | Spearman's rho | -0.218 | -0.072 | 0.047 | -0.180 | -0.207 | -0.179 | -0.162 | -0.134 | 0.005 | 0.032 | -0.075 | -0.334 |
|  | p-value | **0.017** | 0.942 | 0.740 | 0.051 | **0.023** | 0.059 | 0.093 | 0.183 | 0.960 | 0.813 | 0.534 | **<0.001** |
| alpha-beta | Spearman's rho | -0.255 | 0.070 | 0.053 | -0.211 | -0.240 | -0.235 | -0.265 | -0.061 | 0.180 | 0.120 | -0.096 | -0.143 |
|  | p-value | **0.008** | 0.942 | 0.740 | **0.027** | **0.009** | **0.018** | **0.005** | 0.501 | 0.110 | 0.327 | 0.534 | 0.133 |
| alpha-gamma | Spearman's rho | -0.374 | -0.077 | -0.065 | -0.357 | -0.418 | -0.351 | -0.367 | -0.204 | 0.276 | 0.306 | -0.086 | -0.416 |
|  | p-value | **<0.001** | 0.942 | 0.740 | **<0.001** | **<0.001** | **<0.001** | **<0.001** | **0.048** | **0.020** | **<0.001** | 0.534 | **<0.001** |
| beta-gamma | Spearman's rho | -0.358 | -0.006 | 0.026 | -0.373 | -0.427 | -0.370 | -0.344 | -0.225 | 0.233 | 0.265 | -0.100 | -0.448 |
|  | p-value | **<0.001** | 0.952 | 0.853 | **<0.001** | **<0.001** | **<0.001** | **<0.001** | **0.048** | **0.037** | **0.013** | 0.534 | **<0.001** |

P-values are FDR-corrected for Spearman correlation analysis. P-values less than 0.05 are in bold. MMSE = Mini-Mental State Examination, HAMA = Hamilton Anxiety Rating Scale, HAMD = Hamilton Depression Rating Scale, HVLT = Hopkins Verbal Learning Test, STT = Shape trials test. Larger scores on the STT-A or STT-B test indicate that more time is needed to complete the test and suggest poorer executive performance. HC = healthy control, MCI = mild cognitive impairment, AD = Alzheimer's disease.

**Supplementary Table 2**. Correlations between cognitive scale scores andcouplings inregion-of-interest.

|  |  |  | MMSE | HAMA | HAMD | Memory function | | | Language function | | Executive function | | Visual space navigation function | |
| --- | --- | --- | --- | --- | --- | --- | --- | --- | --- | --- | --- | --- | --- | --- |
|  |  |  |  |  |  | HVLT-  Immediate recall | HVLT-  Delayed recall | Logical memory test | Boston naming test | Verbal fluency test | STT-A | STT-B | ROCF  (copy scores) | ROCF  (recall scores) |
|  |  |  |  |  |  |  |  |  |  |  |  |  |  |  |
|  | Left frontal | Spearman's rho | -0.111 | 0.097 | 0.048 | -0.141 | -0.041 | -0.157 | -0.157 | -0.087 | 0.007 | -0.069 | -0.119 | -0.190 |
| Delta-theta |  | p-value | 0.552 | 0.936 | 0.713 | 0.440 | 0.737 | 0.200 | 0.300 | 0.569 | 0.997 | 0.708 | 0.629 | 0.124 |
|  | Left temporal | Spearman's rho | -0.139 | -0.145 | -0.101 | -0.108 | -0.083 | -0.165 | -0.057 | -0.074 | 0.032 | -0.041 | -0.077 | -0.151 |
|  |  | p-value | 0.448 | 0.716 | 0.664 | 0.589 | 0.655 | 0.200 | 0.834 | 0.569 | 0.997 | 0.757 | 0.629 | 0.235 |
|  | Left parietal | Spearman's rho | -0.063 | -0.014 | 0.103 | -0.055 | -0.090 | -0.090 | -0.122 | -0.070 | -0.000 | -0.060 | -0.076 | -0.126 |
|  |  | p-value | 0.588 | 0.955 | 0.664 | 0.618 | 0.655 | 0.618 | 0.386 | 0.569 | 0.997 | 0.708 | 0.629 | 0.312 |
|  | Left occipital | Spearman's rho | -0.048 | 0.006 | 0.045 | -0.043 | -0.062 | -0.030 | -0.003 | 0.039 | -0.127 | -0.230 | -0.081 | -0.048 |
|  |  | p-value | 0.588 | 0.955 | 0.713 | 0.625 | 0.655 | 0.838 | 0.973 | 0.753 | 0.808 | 0.112 | 0.629 | 0.590 |
|  | Right frontal | Spearman's rho | -0.048 | -0.075 | 0.083 | -0.054 | -0.061 | -0.018 | -0.043 | 0.000 | -0.115 | -0.113 | -0.009 | -0.084 |
|  |  | p-value | 0.588 | 0.938 | 0.686 | 0.618 | 0.655 | 0.843 | 0.840 | 0.999 | 0.808 | 0.708 | 0.970 | 0.550 |
|  | Right temporal | Spearman's rho | -0.070 | -0.139 | 0.027 | -0.078 | 0.007 | -0.040 | -0.012 | -0.094 | -0.031 | 0.059 | -0.003 | -0.053 |
|  |  | p-value | 0.488 | 0.716 | 0.762 | 0.618 | 0.936 | 0.838 | 0.973 | 0.569 | 0.997 | 0.708 | 0.970 | 0.590 |
|  | Right parietal | Spearman's rho | -0.187 | 0.028 | 0.043 | -0.157 | -0.222 | -0.211 | -0.216 | -0.115 | 0.052 | 0.098 | -0.101 | -0.265 |
|  |  | p-value | 0.256 | 0.955 | 0.713 | 0.440 | 0.088 | 0.128 | 0.104 | 0.569 | 0.997 | 0.708 | 0.629 | **0.016** |
|  | Right occipital | Spearman's rho | -0.087 | 0.056 | 0.154 | -0.073 | -0.093 | -0.058 | -0.115 | -0.072 | -0.022 | -0.01 | -0.045 | -0.069 |
|  |  | p-value | 0.588 | 0.942 | 0.616 | 0.618 | 0.655 | 0.819 | 0.386 | 0.569 | 0.997 | 0.913 | 0.820 | 0.587 |
| Delta-alpha | Left frontal | Spearman's rho | -0.268 | -0.155 | -0.135 | -0.345 | -0.387 | -0.306 | -0.270 | -0.201 | 0.167 | 0.312 | -0.094 | -0.310 |
|  |  | p-value | **0.004** | 0.357 | 0.492 | **<0.001** | **<0.001** | **<0.001** | **0.005** | **0.035** | 0.121 | **<0.001** | 0.733 | **<0.001** |
|  | Left temporal | Spearman's rho | -0.216 | -0.170 | -0.070 | -0.281 | -0.314 | -0.254 | -0.105 | -0.316 | 0.151 | 0.231 | -0.053 | -0.268 |
|  |  | p-value | **0.017** | 0.357 | 0.691 | **0.001** | **<0.001** | **0.006** | 0.234 | **<0.001** | 0.121 | **0.021** | 0.733 | **0.004** |
|  | Left parietal | Spearman's rho | -0.304 | -0.117 | -0.057 | -0.386 | -0.388 | -0.343 | -0.363 | -0.254 | 0.302 | 0.305 | 0.119 | -0.196 |
|  |  | p-value | **<0.001** | 0.416 | 0.691 | **<0.001** | **<0.001** | **<0.001** | **<0.001** | **0.011** | **<0.001** | **<0.001** | 0.708 | **0.030** |
|  | Left occipital | Spearman's rho | -0.255 | -0.034 | -0.006 | -0.208 | -0.267 | -0.229 | -0.185 | -0.101 | 0.131 | 0.143 | -0.007 | -0.214 |
|  |  | p-value | **0.030** | 0.742 | 0.942 | **0.017** | **0.002** | **0.012** | **0.047** | 0.252 | 0.163 | 0.145 | 0.936 | **0.020** |
|  | Right frontal | Spearman's rho | -0.263 | -0.273 | -0.184 | -0.297 | -0.345 | -0.254 | -0.209 | -0.259 | 0.246 | 0.344 | 0.039 | -0.234 |
|  |  | p-value | **0.004** | 0.056 | 0.280 | **<0.001** | **<0.001** | **0.006** | **0.027** | **0.011** | **0.020** | **<0.001** | 0.752 | **0.013** |
|  | Right temporal | Spearman's rho | -0.207 | -0.131 | 0.058 | -0.269 | -0.250 | -0.100 | -0.109 | -0.163 | 0.152 | 0.071 | 0.065 | -0.169 |
|  |  | p-value | **0.019** | 0.412 | 0.691 | **0.002** | **0.004** | 0.256 | 0.234 | 0.072 | 0.121 | 0.451 | 0.733 | 0.056 |
|  | Right parietal | Spearman's rho | -0.294 | -0.072 | -0.019 | -0.332 | -0.395 | -0.269 | -0.273 | -0.205 | 0.182 | 0.259 | -0.080 | -0.350 |
|  |  | p-value | **<0.001** | 0.619 | 0.942 | **<0.001** | **<0.001** | **0.005** | **0.005** | **0.035** | 0.109 | **0.010** | 0.733 | **<0.001** |
|  | Right occipital | Spearman's rho | -0.247 | -0.063 | -0.073 | -0.322 | -0.323 | -0.220 | -0.222 | -0.182 | 0.114 | 0.205 | -0.198 | -0.270 |
|  |  | p-value | **0.006** | 0.619 | 0.691 | **<0.001** | **<0.001** | **0.014** | **0.022** | 0.051 | 0.203 | **0.037** | 0.192 | **0.004** |
| Delta-beta | Left frontal | Spearman's rho | -0.189 | 0.087 | 0.097 | -0.216 | -0.217 | -0.182 | -0.145 | -0.150 | -0.017 | 0.003 | -0.099 | -0.197 |
|  |  | p-value | 0.080 | 0.800 | 0.561 | 0.056 | **0.026** | 0.101 | 0.128 | 0.198 | 0.967 | 0.973 | 0.656 | 0.050 |
|  | Left temporal | Spearman's rho | -0.102 | 0.090 | 0.052 | -0.165 | -0.121 | -0.137 | -0.179 | -0.252 | 0.129 | 0.041 | -0.050 | -0.012 |
|  |  | p-value | 0.246 | 0.800 | 0.630 | 0.096 | 0.170 | 0.160 | 0.128 | **0.032** | 0.967 | 0.808 | 0.656 | 0.895 |
|  | Left parietal | Spearman's rho | -0.153 | -0.002 | 0.022 | -0.177 | -0.300 | -0.141 | -0.169 | -0.112 | 0.083 | 0.037 | -0.051 | -0.202 |
|  |  | p-value | 0.107 | 0.988 | 0.803 | 0.088 | **<0.001** | 0.160 | 0.128 | 0.326 | 0.967 | 0.808 | 0.656 | 0.050 |
|  | Left occipital | Spearman's rho | -0.163 | -0.020 | 0.074 | -0.147 | -0.206 | -0.162 | -0.139 | -0.082 | 0.024 | -0.070 | -0.123 | -0.172 |
|  |  | p-value | 0.099 | 0.971 | 0.561 | 0.127 | **0.030** | 0.130 | 0.128 | 0.401 | 0.967 | 0.808 | 0.656 | 0.083 |
|  | Right frontal | Spearman's rho | -0.163 | -0.204 | -0.093 | -0.179 | -0.247 | -0.201 | -0.134 | -0.168 | 0.003 | 0.035 | -0.051 | -0.202 |
|  |  | p-value | 0.099 | 0.328 | 0.561 | 0.088 | **0.013** | 0.088 | 0.128 | 0.198 | 0.971 | 0.808 | 0.656 | 0.050 |
|  | Right temporal | Spearman's rho | -0.194 | -0.179 | -0.071 | -0.065 | -0.170 | 0.002 | -0.144 | -0.145 | 0.046 | -0.048 | -0.027 | -0.094 |
|  |  | p-value | 0.080 | 0.328 | 0.561 | 0.465 | 0.072 | 0.985 | 0.128 | 0.198 | 0.967 | 0.808 | 0.765 | 0.329 |
|  | Right parietal | Spearman's rho | -0.222 | 0.039 | 0.120 | -0.222 | -0.275 | -0.214 | -0.202 | -0.098 | 0.057 | 0.095 | -0.054 | -0.217 |
|  |  | p-value | 0.080 | 0.956 | 0.561 | 0.056 | **0.008** | 0.088 | 0.128 | 0.353 | 0.967 | 0.808 | 0.656 | 0.050 |
|  | Right occipital | Spearman's rho | -0.110 | 0.038 | 0.084 | -0.103 | -0.144 | -0.089 | -0.137 | 0.003 | -0.080 | -0.042 | -0.123 | -0.118 |
|  |  | p-value | 0.240 | 0.956 | 0.561 | 0.280 | 0.117 | 0.355 | 0.128 | 0.977 | 0.967 | 0.808 | 0.656 | 0.244 |
| Delta-gamma | Left frontal | Spearman's rho | -0.117 | -0.084 | -0.018 | -0.209 | -0.240 | -0.183 | -0.109 | -0.169 | 0.000 | 0.056 | -0.166 | 0.325 |
|  |  | p-value | 0.183 | 0.792 | 0.954 | **0.027** | **0.009** | **0.049** | 0.289 | 0.108 | 0.998 | 0.627 | 0.472 | **<0.001** |
|  | Left temporal | Spearman's rho | -0.356 | -0.211 | -0.148 | -0.400 | -0.431 | -0.330 | -0.295 | -0.276 | 0.234 | 0.327 | -0.053 | -0.349 |
|  |  | p-value | **<0.001** | 0.320 | 0.720 | **<0.001** | **<0.001** | **<0.001** | **<0.001** | **0.008** | **0.032** | **<0.001** | 0.867 | **<0.001** |
|  | Left parietal | Spearman's rho | -0.230 | -0.044 | -0.024 | -0.199 | -0.236 | -0.254 | -0.208 | -0.152 | 0.071 | 0.071 | 0.072 | -0.248 |
|  |  | p-value | **0.016** | 0.792 | 0.954 | **0.031** | **0.009** | **0.011** | **0.029** | 0.136 | 0.717 | 0.627 | 0.867 | **0.007** |
|  | Left occipital | Spearman's rho | -0.136 | 0.003 | 0.026 | -0.035 | -0.072 | -0.079 | -0.062 | -0.135 | -0.068 | -0.017 | -0.037 | -0.162 |
|  |  | p-value | 0.138 | 0.975 | 0.954 | 0.691 | 0.415 | 0.369 | 0.480 | 0.144 | 0.717 | 0.860 | 0.867 | 0.067 |
|  | Right frontal | Spearman's rho | -0.137 | -0.064 | -0.032 | -0.131 | -0.173 | -0.083 | -0.085 | -0.182 | 0.006 | 0.081 | -0.011 | -0.253 |
|  |  | p-value | 0.138 | 0.792 | 0.954 | 0.155 | 0.057 | 0.369 | 0.382 | 0.101 | 0.998 | 0.627 | 0.905 | **0.006** |
|  | Right temporal | Spearman's rho | -0.342 | -0.134 | -0.058 | -0.326 | -0.398 | -0.345 | -0.357 | -0.275 | 0.256 | 0.227 | -0.067 | -0.372 |
|  |  | p-value | **<0.001** | 0.780 | 0.954 | **<0.001** | **<0.001** | **<0.001** | **<0.001** | **0.008** | **0.032** | 0.060 | 0.867 | **<0.001** |
|  | Right parietal | Spearman's rho | -0.252 | -0.079 | 0.017 | -0.310 | -0.423 | -0.229 | -0.302 | -0.115 | 0.158 | 0.180 | -0.063 | -0.386 |
|  |  | p-value | **0.011** | 0.792 | 0.954 | **<0.001** | **<0.001** | **0.014** | **<0.001** | 0.191 | 0.205 | 0.144 | 0.867 | **<0.001** |
|  | Right occipital | Spearman's rho | -0.194 | 0.041 | -0.005 | -0.211 | -0.278 | -0.230 | -0.217 | -0.140 | -0.001 | 0.061 | -0.027 | -0.239 |
|  |  | p-value | **0.042** | 0.792 | 0.954 | **0.027** | **0.002** | **0.014** | **0.026** | 0.144 | 0.998 | 0.627 | 0.867 | **0.007** |
| Theta-alpha | Left frontal | Spearman's rho | -0.192 | -0.189 | -0.140 | -0.240 | -0.229 | -0.185 | -0.124 | -0.148 | 0.115 | 0.214 | -0.036 | -0.252 |
|  |  | p-value | 0.056 | 0.264 | 0.460 | **0.016** | **0.029** | 0.070 | 0.466 | 0.147 | 0.336 | 0.077 | 0.958 | **0.016** |
|  | Left temporal | Spearman's rho | -0.121 | -0.144 | -0.116 | -0.040 | -0.077 | -0.054 | 0.055 | -0.179 | 0.064 | 0.049 | -0.081 | -0.108 |
|  |  | p-value | 0.166 | 0.264 | 0.460 | 0.652 | 0.386 | 0.545 | 0.671 | 0.084 | 0.639 | 0.601 | 0.958 | 0.221 |
|  | Left parietal | Spearman's rho | -0.202 | -0.144 | -0.105 | -0.326 | -0.257 | -0.206 | -0.201 | -0.251 | 0.175 | 0.169 | 0.009 | -0.157 |
|  |  | p-value | 0.053 | 0.264 | 0.460 | **<0.001** | **0.024** | 0.067 | 0.176 | **0.032** | 0.200 | 0.144 | 0.958 | 0.101 |
|  | Left occipital | Spearman's rho | -0.169 | 0.013 | 0.015 | -0.138 | -0.162 | -0.100 | -0.048 | -0.080 | 0.017 | 0.069 | -0.008 | -0.193 |
|  |  | p-value | 0.071 | 0.900 | 0.862 | 0.133 | 0.074 | 0.294 | 0.671 | 0.364 | 0.850 | 0.601 | 0.958 | 0.056 |
|  | Right frontal | Spearman's rho | -0.208 | -0.273 | -0.121 | -0.190 | -0.185 | -0.196 | -0.093 | -0.187 | 0.112 | 0.244 | 0.015 | -0.141 |
|  |  | p-value | 0.053 | 0.056 | 0.460 | **0.048** | 0.056 | 0.067 | 0.466 | 0.084 | 0.336 | 0.072 | 0.958 | 0.126 |
|  | Right temporal | Spearman's rho | -0.171 | -0.169 | -0.084 | -0.201 | -0.208 | -0.120 | -0.096 | -0.114 | 0.159 | 0.133 | 0.005 | -0.177 |
|  |  | p-value | 0.071 | 0.264 | 0.472 | **0.044** | **0.034** | 0.237 | 0.466 | 0.260 | 0.200 | 0.250 | 0.958 | 0.070 |
|  | Right parietal | Spearman's rho | -0.273 | -0.104 | -0.081 | -0.253 | -0.223 | -0.203 | -0.113 | -0.179 | 0.188 | 0.203 | -0.050 | -0.253 |
|  |  | p-value | **0.016** | 0.423 | 0.472 | **0.016** | **0.029** | 0.067 | 0.466 | 0.084 | 0.200 | 0.077 | 0.958 | **0.016** |
|  | Right occipital | Spearman's rho | -0.158 | -0.029 | -0.053 | -0.154 | -0.171 | -0.119 | -0.027 | -0.096 | 0.027 | 0.057 | -0.119 | -0.198 |
|  |  | p-value | 0.081 | 0.891 | 0.621 | 0.107 | 0.069 | 0.237 | 0.760 | 0.314 | 0.850 | 0.601 | 0.958 | 0.056 |
| Theta-beta | Left frontal | Spearman's rho | -0.074 | 0.043 | 0.108 | -0.112 | -0.123 | -0.074 | -0.073 | -0.100 | -0.122 | -0.119 | -0.073 | -0.045 |
|  |  | p-value | 0.615 | 0.909 | 0.508 | 0.771 | 0.370 | 0.808 | 0.781 | 0.345 | 0.592 | 0.576 | 0.645 | 0.609 |
|  | Left temporal | Spearman's rho | -0.044 | 0.013 | 0.151 | -0.069 | 0.018 | 0.013 | -0.089 | -0.123 | -0.009 | -0.187 | -0.057 | 0.087 |
|  |  | p-value | 0.615 | 0.939 | 0.508 | 0.771 | 0.841 | 0.882 | 0.781 | 0.345 | 0.918 | 0.368 | 0.645 | 0.436 |
|  | Left parietal | Spearman's rho | -0.050 | -0.078 | 0.030 | -0.073 | -0.152 | -0.033 | -0.032 | -0.128 | -0.017 | -0.058 | -0.089 | -0.107 |
|  |  | p-value | 0.615 | 0.909 | 0.837 | 0.771 | 0.336 | 0.808 | 0.781 | 0.345 | 0.918 | 0.611 | 0.645 | 0.406 |
|  | Left occipital | Spearman's rho | -0.088 | -0.052 | 0.013 | -0.056 | -0.069 | -0.052 | -0.045 | -0.118 | -0.031 | -0.116 | -0.149 | -0.135 |
|  |  | p-value | 0.615 | 0.909 | 0.879 | 0.771 | 0.579 | 0.808 | 0.781 | 0.345 | 0.918 | 0.576 | 0.645 | 0.406 |
|  | Right frontal | Spearman's rho | -0.127 | -0.133 | 0.043 | -0.044 | -0.071 | -0.136 | -0.025 | -0.104 | -0.117 | -0.091 | -0.060 | -0.125 |
|  |  | p-value | 0.576 | 0.800 | 0.836 | 0.771 | 0.579 | 0.488 | 0.781 | 0.345 | 0.592 | 0.611 | 0.645 | 0.406 |
|  | Right temporal | Spearman's rho | -0.108 | -0.152 | -0.081 | -0.033 | -0.117 | -0.037 | -0.185 | -0.086 | 0.094 | 0.021 | -0.051 | -0.101 |
|  |  | p-value | 0.576 | 0.800 | 0.568 | 0.771 | 0.370 | 0.808 | 0.280 | 0.377 | 0.592 | 0.823 | 0.645 | 0.406 |
|  | Right parietal | Spearman's rho | -0.213 | -0.008 | 0.103 | -0.189 | -0.195 | -0.188 | -0.110 | -0.127 | 0.094 | 0.068 | -0.017 | -0.186 |
|  |  | p-value | 0.112 | 0.939 | 0.508 | 0.256 | 0.216 | 0.256 | 0.781 | 0.345 | 0.592 | 0.611 | 0.852 | 0.280 |
|  | Right occipital | Spearman's rho | -0.054 | 0.045 | 0.100 | -0.026 | -0.027 | -0.035 | -0.049 | 0.012 | -0.079 | -0.078 | -0.090 | -0.068 |
|  |  | p-value | 0.615 | 0.909 | 0.508 | 0.771 | 0.841 | 0.808 | 0.781 | 0.890 | 0.610 | 0.611 | 0.645 | 0.507 |
| Theta-gamma | Left frontal | Spearman's rho | -0.120 | -0.148 | 0.018 | -0.180 | -0.221 | -0.157 | -0.121 | -0.133 | 0.015 | 0.020 | -0.147 | -0.303 |
|  |  | p-value | 0.194 | 0.403 | 0.834 | 0.080 | **0.024** | 0.099 | 0.227 | 0.238 | 0.867 | 0.887 | 0.380 | **<0.001** |
|  | Left temporal | Spearman's rho | -0.322 | -0.151 | -0.043 | -0.318 | -0.332 | -0.298 | -0.183 | -0.270 | 0.204 | 0.252 | -0.039 | -0.299 |
|  |  | p-value | **<0.001** | 0.403 | 0.834 | **<0.001** | **<0.001** | **<0.001** | 0.092 | **0.008** | 0.088 | **0.032** | 0.781 | **<0.001** |
|  | Left parietal | Spearman's rho | -0.173 | -0.105 | -0.023 | -0.147 | -0.157 | -0.221 | -0.153 | -0.131 | 0.023 | 0.018 | 0.025 | -0.210 |
|  |  | p-value | 0.075 | 0.626 | 0.834 | 0.147 | 0.100 | **0.024** | 0.133 | 0.238 | 0.867 | 0.887 | 0.781 | **0.019** |
|  | Left occipital | Spearman's rho | -0.107 | -0.012 | 0.113 | -0.018 | 0.004 | -0.062 | -0.049 | -0.011 | -0.100 | -0.067 | -0.058 | -0.183 |
|  |  | p-value | 0.224 | 0.910 | 0.794 | 0.841 | 0.967 | 0.552 | 0.580 | 0.897 | 0.448 | 0.854 | 0.781 | **0.038** |
|  | Right frontal | Spearman's rho | -0.148 | -0.019 | 0.105 | -0.066 | -0.069 | -0.050 | -0.078 | -0.079 | -0.097 | -0.059 | -0.035 | -0.249 |
|  |  | p-value | 0.120 | 0.910 | 0.794 | 0.519 | 0.498 | 0.574 | 0.434 | 0.425 | 0.448 | 0.854 | 0.781 | **0.006** |
|  | Right temporal | Spearman's rho | -0.405 | -0.158 | -0.091 | -0.344 | -0.389 | -0.313 | -0.362 | -0.286 | 0.266 | 0.247 | -0.153 | -0.453 |
|  |  | p-value | **<0.001** | 0.403 | 0.794 | **<0.001** | **<0.001** | **<0.001** | **<0.001** | **<0.001** | **0.024** | **0.032** | 0.380 | **<0.001** |
|  | Right parietal | Spearman's rho | -0.213 | 0.020 | 0.072 | -0.270 | -0.315 | -0.236 | -0.254 | -0.127 | 0.111 | 0.095 | -0.077 | -0.324 |
|  |  | p-value | **0.037** | 0.910 | 0.794 | **0.005** | **<0.001** | **0.019** | **0.012** | 0.238 | 0.448 | 0.829 | 0.781 | **<0.001** |
|  | Right occipital | Spearman's rho | -0.186 | 0.026 | 0.060 | -0.141 | -0.193 | -0.159 | -0.176 | -0.106 | -0.025 | 0.013 | -0.028 | -0.226 |
|  |  | p-value | 0.066 | 0.910 | 0.794 | 0.147 | **0.045** | 0.099 | 0.092 | 0.307 | 0.867 | 0.887 | 0.781 | **0.013** |
| Alpha-beta | Left frontal | Spearman's rho | -0.207 | 0.179 | 0.060 | -0.229 | -0.218 | -0.262 | -0.209 | -0.114 | 0.092 | 0.055 | -0.082 | -0.106 |
|  |  | p-value | **0.045** | 0.332 | 0.794 | **0.018** | **0.035** | **0.008** | **0.023** | 0.523 | 0.461 | 0.752 | 0.602 | 0.309 |
|  | Left temporal | Spearman's rho | -0.184 | -0.006 | -0.083 | -0.133 | -0.068 | -0.179 | -0.129 | -0.184 | 0.093 | -0.015 | -0.106 | -0.026 |
|  |  | p-value | **0.047** | 0.950 | 0.710 | 0.176 | 0.440 | 0.056 | 0.144 | 0.196 | 0.461 | 0.981 | 0.602 | 0.772 |
|  | Left parietal | Spearman's rho | -0.163 | 0.059 | -0.029 | -0.257 | -0.272 | -0.207 | -0.226 | -0.173 | 0.186 | 0.148 | -0.126 | -0.149 |
|  |  | p-value | 0.071 | 0.950 | 0.895 | **0.018** | **0.008** | **0.036** | **0.016** | 0.196 | 0.196 | 0.580 | 0.602 | 0.245 |
|  | Left occipital | Spearman's rho | -0.189 | 0.053 | 0.012 | -0.163 | -0.123 | -0.190 | -0.170 | -0.025 | 0.066 | 0.002 | -0.162 | -0.121 |
|  |  | p-value | **0.047** | 0.950 | 0.895 | 0.102 | 0.186 | **0.048** | 0.062 | 0.801 | 0.461 | 0.981 | 0.528 | 0.309 |
|  | Right frontal | Spearman's rho | -0.305 | 0.072 | 0.081 | -0.238 | -0.184 | -0.266 | -0.236 | -0.063 | 0.138 | 0.066 | -0.078 | -0.168 |
|  |  | p-value | **<0.001** | 0.950 | 0.710 | **0.018** | 0.074 | **0.008** | **0.016** | 0.685 | 0.328 | 0.752 | 0.602 | 0.224 |
|  | Right temporal | Spearman's rho | -0.188 | -0.218 | -0.103 | -0.078 | -0.169 | -0.126 | -0.255 | -0.022 | 0.078 | 0.054 | 0.016 | -0.096 |
|  |  | p-value | **0.047** | 0.272 | 0.710 | 0.378 | 0.088 | 0.175 | **0.016** | 0.801 | 0.461 | 0.752 | 0.900 | 0.319 |
|  | Right parietal | Spearman's rho | -0.286 | 0.020 | 0.097 | -0.227 | -0.290 | -0.278 | -0.254 | -0.058 | 0.176 | 0.137 | 0.011 | -0.184 |
|  |  | p-value | **<0.001** | 0.950 | 0.710 | **0.018** | **<0.001** | **0.008** | **0.016** | 0.685 | 0.196 | 0.580 | 0.900 | 0.224 |
|  | Right occipital | Spearman's rho | -0.147 | 0.026 | 0.024 | -0.090 | -0.146 | -0.116 | -0.232 | -0.058 | 0.069 | 0.067 | -0.025 | -0.114 |
|  |  | p-value | 0.093 | 0.950 | 0.895 | 0.350 | 0.131 | 0.189 | **0.016** | 0.685 | 0.461 | 0.752 | 0.900 | 0.309 |
| Alpha-gamma | Left frontal | Spearman's rho | -0.265 | -0.120 | -0.062 | -0.294 | -0.360 | -0.338 | -0.301 | -0.189 | 0.193 | 0.191 | -0.168 | -0.373 |
|  |  | p-value | **0.003** | 0.898 | 0.941 | **<0.001** | **<0.001** | **<0.001** | **<0.001** | 0.059 | **0.048** | **0.047** | 0.224 | **<0.001** |
|  | Left temporal | Spearman's rho | -0.302 | -0.154 | -0.097 | -0.329 | -0.315 | -0.275 | -0.301 | -0.203 | 0.229 | 0.271 | -0.143 | -0.245 |
|  |  | p-value | **<0.001** | 0.898 | 0.941 | **<0.001** | **<0.001** | **0.003** | **<0.001** | 0.059 | **0.027** | **0.012** | 0.280 | **0.006** |
|  | Left parietal | Spearman's rho | -0.255 | 0.013 | -0.039 | -0.274 | -0.281 | -0.312 | -0.273 | -0.133 | 0.196 | 0.278 | -0.014 | -0.227 |
|  |  | p-value | **0.003** | 0.898 | 0.941 | **0.002** | **0.001** | **<0.001** | **0.003** | 0.151 | **0.048** | **0.012** | 0.970 | 0.101 |
|  | Left occipital | Spearman's rho | -0.215 | -0.052 | -0.033 | -0.170 | -0.177 | -0.197 | -0.101 | -0.174 | 0.075 | 0.121 | -0.058 | -0.251 |
|  |  | p-value | **0.013** | 0.898 | 0.941 | 0.053 | **0.044** | **0.025** | 0.252 | 0.064 | 0.401 | 0.197 | 0.821 | **0.005** |
|  | Right frontal | Spearman's rho | -0.315 | 0.018 | 0.007 | -0.291 | -0.255 | -0.228 | -0.254 | -0.195 | 0.181 | 0.247 | -0.063 | -0.284 |
|  |  | p-value | **<0.001** | 0.898 | 0.941 | **<0.001** | **0.003** | **0.010** | **0.003** | 0.059 | 0.056 | **0.020** | 0.821 | **0.002** |
|  | Right temporal | Spearman's rho | -0.322 | -0.068 | -0.032 | -0.268 | -0.286 | -0.316 | -0.395 | -0.237 | 0.265 | 0.223 | -0.181 | -0.318 |
|  |  | p-value | **<0.001** | 0.898 | 0.941 | **0.002** | **<0.001** | **<0.001** | **<0.001** | 0.056 | **0.016** | **0.027** | 0.224 | **<0.001** |
|  | Right parietal | Spearman's rho | -0.316 | -0.023 | -0.031 | -0.315 | -0.434 | -0.291 | -0.330 | -0.089 | 0.255 | 0.238 | -0.003 | -0.375 |
|  |  | p-value | **<0.001** | 0.898 | 0.941 | **<0.001** | **<0.001** | **<0.001** | **<0.001** | 0.316 | **0.016** | **0.020** | 0.970 | **<0.001** |
|  | Right occipital | Spearman's rho | -0.358 | -0.091 | -0.015 | -0.322 | -0.394 | -0.303 | -0.362 | -0.183 | 0.168 | 0.206 | -0.034 | -0.346 |
|  |  | p-value | **<0.001** | 0.898 | 0.941 | **<0.001** | **<0.001** | **<0.001** | **<0.001** | 0.059 | 0.069 | **0.036** | 0.936 | **<0.001** |
| Beta-gamma | Left frontal | Spearman's rho | -0.197 | -0.055 | -0.066 | -0.257 | -0.319 | -0.285 | -0.222 | -0.168 | 0.087 | 0.099 | -0.149 | -0.349 |
|  |  | p-value | **0.024** | 0.903 | 0.750 | **0.003** | **<0.001** | **<0.001** | **0.013** | 0.064 | 0.335 | 0.292 | 0.280 | **<0.001** |
|  | Left temporal | Spearman's rho | -0.243 | -0.157 | -0.064 | -0.300 | -0.326 | -0.225 | -0.228 | -0.220 | 0.190 | 0.212 | -0.080 | -0.181 |
|  |  | p-value | **0.006** | 0.903 | 0.750 | **<0.001** | **<0.001** | **0.011** | **0.012** | **0.043** | 0.053 | **0.046** | 0.586 | **0.040** |
|  | Left parietal | Spearman's rho | -0.303 | 0.057 | 0.026 | -0.338 | -0.367 | -0.407 | -0.337 | -0.141 | 0.236 | 0.220 | -0.005 | -0.346 |
|  |  | p-value | **<0.001** | 0.903 | 0.777 | **<0.001** | **<0.001** | **<0.001** | **<0.001** | 0.109 | **0.032** | **0.046** | 0.959 | **<0.001** |
|  | Left occipital | Spearman's rho | -0.340 | 0.017 | 0.104 | -0.305 | -0.321 | -0.300 | -0.302 | -0.197 | 0.195 | 0.241 | -0.143 | -0.375 |
|  |  | p-value | **<0.001** | 0.903 | 0.750 | **<0.001** | **<0.001** | **<0.001** | **<0.001** | 0.050 | 0.053 | **0.040** | 0.280 | **<0.001** |
|  | Right frontal | Spearman's rho | -0.284 | -0.028 | -0.031 | -0.233 | -0.243 | -0.214 | -0.170 | -0.211 | 0.113 | 0.190 | -0.008 | -0.342 |
|  |  | p-value | **<0.001** | 0.903 | 0.777 | **0.008** | **0.005** | **0.015** | 0.053 | **0.043** | 0.240 | 0.056 | 0.959 | **<0.001** |
|  | Right temporal | Spearman's rho | -0.354 | -0.016 | -0.099 | -0.257 | -0.264 | -0.333 | -0.316 | -0.234 | 0.201 | 0.143 | -0.182 | -0.353 |
|  |  | p-value | **<0.001** | 0.903 | 0.750 | **0.003** | **0.002** | **<0.001** | **<0.001** | **0.043** | 0.053 | 0.145 | 0.280 | **<0.001** |
|  | Right parietal | Spearman's rho | -0.343 | 0.013 | 0.080 | -0.389 | -0.474 | -0.353 | -0.383 | -0.177 | 0.256 | 0.273 | -0.066 | -0.428 |
|  |  | p-value | **<0.001** | 0.903 | 0.750 | **<0.001** | **<0.001** | **<0.001** | **<0.001** | 0.059 | **0.032** | **0.024** | 0.609 | **<0.001** |
|  | Right occipital | Spearman's rho | -0.251 | -0.066 | -0.025 | -0.333 | -0.424 | -0.338 | -0.356 | -0.185 | 0.177 | 0.195 | -0.104 | -0.405 |
|  |  | p-value | **0.005** | 0.903 | 0.777 | **<0.001** | **<0.001** | **<0.001** | **<0.001** | 0.056 | 0.063 | 0.056 | 0.476 | **<0.001** |

P-values are FDR-corrected for Spearman correlation analysis. P-values less than 0.05 are in bold. MMSE = Mini-Mental State Examination, HAMA = Hamilton Anxiety Rating Scale, HAMD = Hamilton Depression Rating Scale, HVLT = Hopkins Verbal Learning Test, STT = Shape trials test. Larger scores on the STT-A or STT-B test indicate that more time is needed to complete the test and suggest poorer executive performance. HC = healthy control, MCI = mild cognitive impairment, AD = Alzheimer's disease.
